# Innovative Bio-Based Organic UV-A and Blue Light Filters from Meldrum’s Acid

**DOI:** 10.3390/molecules25092178

**Published:** 2020-05-06

**Authors:** Cédric Peyrot, Matthieu M. Mention, Fanny Brunissen, Patrick Balaguer, Florent Allais

**Affiliations:** 1 URD Agro-Biotechnologies Industrielles (ABI), CEBB, AgroParisTech, 51110 Pomacle, France; cedric.peyrot@agroparistech.fr (C.P.); matthieu.mention@agroparistech.fr (M.M.M.); fanny.brunissen@agroparistech.fr (F.B.); 2 IRCM, Inserm, Univ Montpellier, ICM, 208 Avenue des Apothicaires, CEDEX 5, 34298 Montpellier, France; patrick.balaguer@icm.unicancer.fr

**Keywords:** photoprotection, bio-based chemicals, Meldrum’s acid, UV-A, blue light, organic UV filters

## Abstract

Faced with the ban of some organic UV filters such as octinoxate or avobenzone, especially in Hawaii, it became essential to offer new alternatives that are both renewable and safe for humans and the environment. In this context, a class of bio-based molecules displaying interesting UV filter properties and great (photo)stability has been developed from Meldrum’s acid and bio-based and synthetic *p*-hydroxycinnamic acids, furans and pyrroles. Moreover, *p*-hydroxycinnamic acid-based Meldrum’s derivatives possess valuable secondary activities sought by the cosmetic industry such as antioxidant and anti-tyrosinase properties. The evaluation of the properties of mixture of judiciously chosen Meldrum’s acid derivatives highlighted the possibility to modulate secondary activity while maintaining excellent UV protection. Meldrum’s acid derivatives are not only competitive when benchmarked against organic filters currently on the market (i.e., avobenzone), but they also do not exhibit any endocrine disruption activity.

## 1. Introduction

While solar light is biologically crucial for life on Earth, significant exposure to radiations can have negative effects on the skin [1]. It has been widely shown that UV-B (290–320 nm) and UV-A (320–400 nm) penetrate the different skin barriers and lead to the formation of reactive oxygen species (ROS) [2], inducing oxidative damage resulting in lipid membrane alteration [3,4] or DNA mutations [5,6]. Up until recently, blue light (400–500 nm) irradiation effects were poorly investigated. Even though its impact on the sleep cycle, memory, and retina is clearly highlighted, its impact on skin cells is still under examination [7,8,9].

**Scheme 1:**
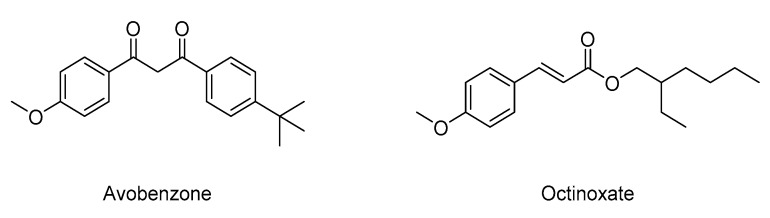
Structures of two common commercial organic filters used in sunscreens.

To protect oneself from these harmful radiations, a wide range of sunscreen lotions and cosmetic creams are available. Some contain organic filters (i.e., avobenzone, octinoxate, benzophenone, or octocrylene) that are increasingly criticized by consumers and countries due to their environmental impact [10,11,12]. Indeed, Hawaii recently issued a bill, effective on 1 January 2021, against the use and sale of sunscreen containing avobenzone or octinoxate [13]. These molecules have been the subject of many studies in the last few years to demonstrate their negative impact on corals [14,15,16]. Indeed, avobenzone and octinoxate (Scheme 1) are two ingredients contributing to coral bleaching; if they do not directly kill corals, they put them under significant stress and subject them to increased mortality levels.

Moreover, the (photo)degradation of these products in water promotes the formation of chlorinated phenols well-known to be mostly toxic [17]. Endocrine disruption related to these products has also been highlighted for several years [18,19] and repeated exposures may induce serious developmental effects on reproductive organs and the central nervous system. Still, too few alternatives are proposed to reduce the use of this type of organic filter. The most common are mineral filters such as titanium dioxide or zinc oxide, which are also widely criticized [20,21]. Indeed, nanoparticles may induce inflammatory diseases or exacerbate respiratory allergies and asthma [22]. They are also involved in cardiovascular diseases and promote certain lung cancers [23]. Although the existing UV filters regulatory lists greatly limits the use of new filters, particularly in China, it is essential to offer new organic UV-A and UV-B filters able to protect humans against the harmful effects of the sun while reducing environmental and public health risks.

Conjugated or aromatic compounds are known for their abilities to absorb specific wavelengths, especially ultraviolet ones [24,25,26]. Some of them are bio-sourced or biodegradable (i.e., phenolic and furfural derivatives) and less harmful. Lignin-derived phenolic compounds have been recently used as starting material to develop a new range of UV filters [25,26,27]. It has been shown that wavelengths absorption range can be modulated by modifying the conjugation system through the functionalization of these phenolics [24,28].

In this study, we focus on the coupling between Meldrum’s acid and various conjugated bio-based monomers, notably phenolics and furans, using Knoevenagel condensation in order to modulate the UV absorption band. This synthetic strategy resulted in compounds exhibiting conjugation throughout their backbone and high steric hinderance on the β position, two factors that have been shown in recent studies as important parameters to obtain anti-UV properties [24,25]. The anti-UV coverage and the photostability under UV irradiation of each compound were evaluated and benchmarked against avobenzone to identify the most competitive compounds. In addition to the capacity of the synthesized molecules to act as UV filters, we also investigated whether they could be used to counteract two negative effects of UV radiations on the human skin, namely the formation of radical ROS and hyperpigmentation. Compounds were thus assessed for their antiradical and tyrosinase inhibition potentials. Finally, endocrine disruption assays determined potential health risks associated to these compounds.

## 2. Results

### 2.1. Sustainable Synthesis

**Scheme 2:**
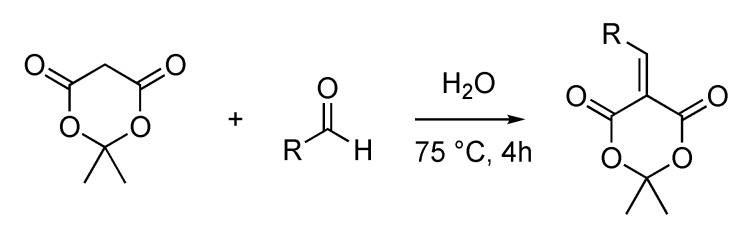
Knoevenagel condensation between Meldrum’s acid and aldehyde in water.

Meldrum’s acid, which results from the condensation of acetone and malonic acid, a bio-based occurring organic acid, possesses a specific reactivity thanks to its highly acidic proton in α of the two esters functions. Knoevenagel reaction resulting from the condensation between Meldrum’s acid and an aldehyde is widely described in the literature and usually does not require the use of a base [29,30]. Different reaction conditions have been investigated in the presence of ionic liquids [31] or in PEG-400 [32]. Nevertheless, the simplest and the greenest synthetic procedure remains the reaction in water as the compounds precipitate in water and can be isolated by a simple filtration, or otherwise they are simply obtained by liquid/liquid extraction, thus avoiding the use of expensive and waste-generating chromatography (Scheme 2) [33]. In order to offer renewable and bio-based products, we oriented our choice for aldehydes towards widely used *p*-hydroxybenzaldehyde (i.e., vanillin) and furanic compounds (i.e., furfural, obtained by dehydration of xyloses). We also chose synthetic aldehydes for a more fundamental purpose (i.e., pyrroles). Through this straightforward and sustainable synthetic procedure, compounds are obtained in high purity and yield, similar to those described in the literature (49%–90%) [33].

**Scheme 3:**
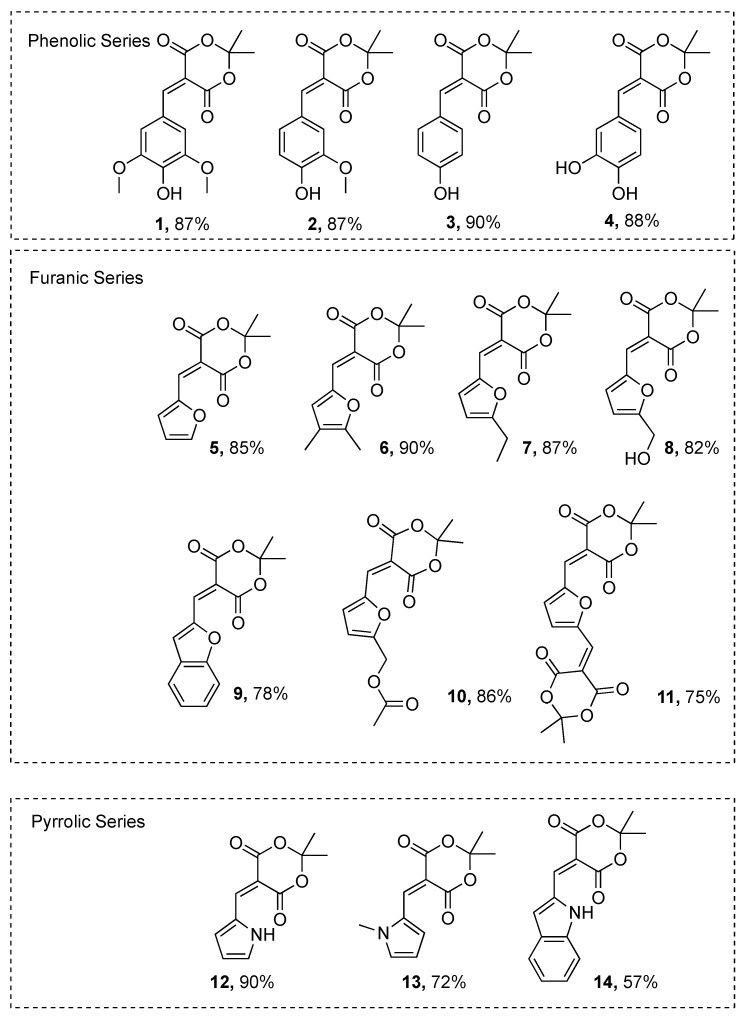
Molecules resulting from the Knoevenagel reaction between Meldrum’s acid and corresponding aldehydes.

Thanks to this simple and sustainable methodology, we populated a library of 14 molecules presenting different conjugation systems (Scheme 3). All of the chemical structures were confirmed by ^1^H- & ^13^C-NMR, and HRMS analyses (see Supporting Information for spectra).

### 2.2. UV Properties and Photostability

**Figure 1:**
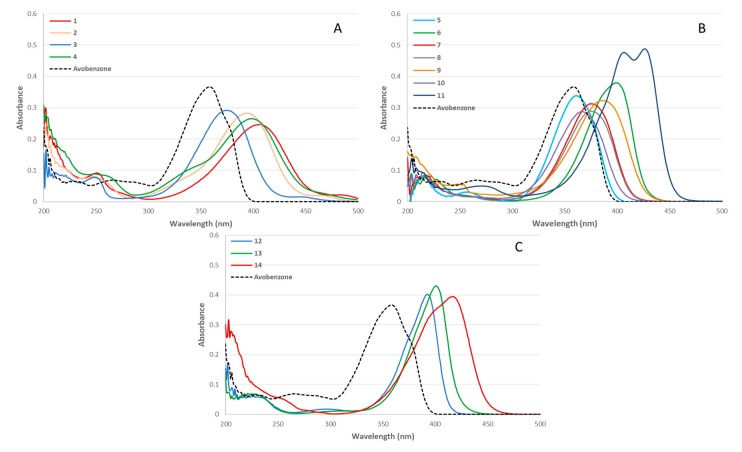
UV–Vis spectra for phenolic series (**A**), furanic series (**B**), and pyrrolic series (**C**), (Concentration = 10 µmol.L^−1^ in ethanol).

In order to evaluate the UV properties of each compound, a UV–Vis spectrum at 10 µmol.L^−1^ in ethanol was performed. With regards to the maximum absorption wavelengths obtained for compounds **1−14** (361–426 nm), their spectra were compared with that of avobenzone (UV-A, λ_max_ = 357 nm) rather than that of octinoxate (UV-B, λ_max_ = 310 nm) (Figure 1A–C).

Very similar spectra were obtained for the phenolic series with absorbance ranging from 300 nm to 475 nm (Figure 1A). The wavelength coverage for the simplest phenolic molecule (**3**) is close to that of avobenzone, but with a lower absorbance. Substituting the phenol moiety with auxochromes (-OH, OMe) resulted in a bathochromic shift of the maximum absorbance, leading to a wavelength coverage that includes part of the UV-A and blue light regions (**1**, **2**, and **4**). Switching from coumaric (**3**) to furan moiety (**5**) allowed to get a λ_max_ (361 nm) closer to that of avobenzone (357 nm). Results for the furanic series (Figure 1B) show a bathochromic shift alike the phenolic series, depending on the electronic effects of the substituents on the furan moiety. With just one substituent on the aromatic ring (**7**, **8**, and **10**), only a small blue shift is observed regardless of the substituent complexity. Introducing a second group on the furan ring leads to a further shift toward blue light that depends on the nature of the substituent and its impact on the conjugation. As expected, the presence of a benzene ring on compound (**9**) conjugated to the furan moiety further expands the π-system and results in a shift further into the blue light spectrum, but without a significant change in absorbance. An increase of the maximal absorbance can be observed with a significant increase of the conjugation on the furan moiety (**11**). However, intermediate absorbance values and blue light shifts are obtained with two unconjugated and not bulky substituents (**6**) (i.e., methyl groups). Finally, the anti-UV properties of the pyrrolic moiety were investigated (Figure 1C). Compared to coumaric (**3**) and furan (**5**), the presence of a simple pyrrole ring (**12**) afforded an absorbance at the frontier between UV-A and blue light regions (λ_max_ = 393 nm). As seen with the previous two series, substitution on the aromatic ring (**13**) provided a bathochromic shift, accentuated with a higher conjugation (**14**), without a significant modification of the maximal absorbance. The study of the structure–activity relationship on the different aromatic moieties highlights the fact that UV properties of such molecules can be fine-tuned by judiciously adjusting the nature of the substituents and conjugation system.

**Figure 2:**
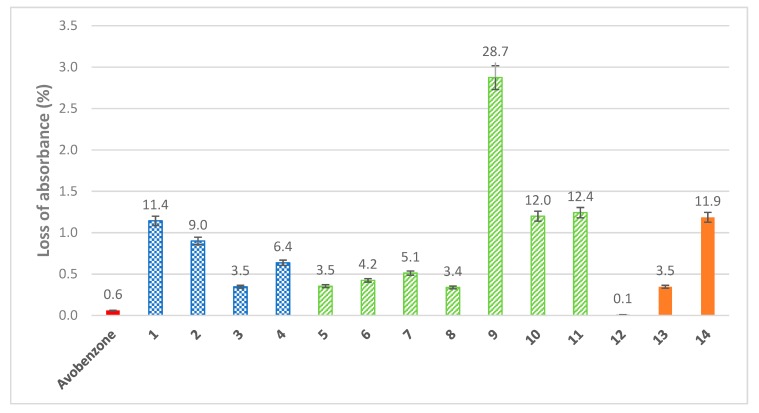
Loss of absorbance for phenolic series (blue checked pattern), furanic series (green stripe), and pyrrolic series (orange), after UV irradiation for 60 min, compared with avobenzone (concentration = 10 μmol.L^−1^ in ethanol).

Beyond the wavelength coverage and absorbance intensity, the photostability (i.e., loss of absorbance over time of exposure) of molecules induced by UV irradiation is of importance when looking at potential UV filters. In order to assess the photostability of synthesized molecules, solutions in ethanol (C = 1 mmol.L^−1^) were irradiated for 60 min under UV light (λ = 300 nm, P = 8.32 W/m^2^, stirring, T = 35 °C). The absorbance of the resulting solutions was compared to that of the non-irradiated solutions (t = 0) (Figure 2).

Discussions with various cosmetic manufacturers have led us to set the loss of absorbance limit to less than 5% in order to properly match commercial UV filters specifications. In such conditions, the reference (avobenzone) underwent only a slight loss of absorbance (0.6%). When comparing the simplest structure of each series (**3**, **5**, and **12**), all three are below the 5% threshold. There is no difference between the coumaric moiety (**3**) and the furanic one (**5**) as the two molecules ended up with a similar loss of absorbance of 3.5%. On the other hand, the introduction of the pyrrole ring (**12**) drastically reduced the loss of absorbance (0.1%), making **12** a more stable filter than avobenzone (0.6%). It is noteworthy to mention that an in-depth kinetic study of the photostability of all compounds upon UV-A/UV-B irradiation, as well as the structural elucidation of the potential photoproducts, will be performed and reported in due course.

In terms of structure–activity relationship, an increase of substitution on the aromatic rings leads to lower stability, as highlighted in the phenolic series where the loss of absorbance of the four molecules studied can be sorted with regards to their substitution on the aromatic ring (coumaric < caffeic < ferulic < sinapic). For both furanic series (**9** and **11**) and pyrrolic series (**14**), an increase of conjugation, either with a benzene ring (**9** and **14**) or through a carbon–carbon double bond (**11**), led to a higher loss of absorbance. The same conclusion can be drawn for the introduction of a sensitive functional group such as an ester (**10**). On the contrary, introduction of a primary alcohol in the furan moiety (**8**) did not result in a higher loss of absorbance as one could have expected with the aforementioned observations, but in a slightly lower loss of absorbance compared to its alkyl analog (**7**). As with absorbance and wavelength coverage, photostability of the molecules is highly impacted by the extent of conjugation, the latter having a negative impact.

### 2.3. Antiradical Properties

Reactive oxygen species (ROS) resulting from the reduction of molecular oxygen are naturally produced via biochemical reactions in the body or from exogenous stimulation and play a role in cell signaling and synaptic plasticity [34]. Despite their natural presence, they can also be harmful when the organism is not able to regulate their level through endogenous antioxidants. Thereby, high concentrations of ROS, which can result from overexposure to the sun for example, have been linked to skin photoaging, inflammations, oxidative stress, or skin carcinogenesis [1]. To limit these harmful effects, antioxidants are conventionally added to sunscreen formulations. The most commonly used molecules are butylated hydroxyanisole (BHA) and butylated hydroxytoluene (BHT). If the novel filters developed herein exhibited antiradical properties in addition to UV absorption, they would protect the skin from UV/blue light radiation while preventing the negative consequences of such exposure by regulating the quantity of ROS generated, without having to add any other compound in the formulation. To determine the potential of the synthesized molecules as antiradicals, their EC_50_ value (half maximal effective concentration), defined as the quantity (in nmol) of antiradical compound needed to reduce 50% of free radicals, was evaluated. The lower the EC_50_ value, the better the antiradical. Tests were performed by using DPPH (2,2-diphenyl-1-picrylhydrazyl) as a free radical and monitoring its disappearance, results are shown in Table 1.

Antiradical properties for the phenolic series have already been described and are in accordance with those measured [33]. Within the furanic and pyrrolic series, only three derivatives demonstrate antiradical activity (**5**, **8**, and **11**) but with poor EC_50_ values (>80 nmol). Of all the synthesized compounds, as expected, only those possessing a phenol moiety exhibited antiradical properties, with only **1** and **4** being competitive against the two commercial references.

### 2.4. Tyrosinase Inhibition

In addition of the negative effects cited before, excessive sun exposure can also induce pigment spots, called age spots, on the exposed skin areas [35]. This hyperpigmentation is due to an abnormal production of melanin, a pigment present in human skin cells that is initially responsible for tanning to protect the skin against UV radiations. This pigment is produced from tyrosine by the action of tyrosinase during melanogenesis. Pigmentary disorders result mainly from an overproduction of melanin due to cell degeneration linked to high sun exposure. One way to limit the appearance of these age spots is to inhibit tyrosinase activity, thus locally reducing the production of melanin and decreasing pigmentation. Tyrosinase inhibition is widely described in the literature [36,37,38,39,40]. In tubo inhibition tests can be performed using a fungal tyrosinase to mimic human tyrosinase activity, making it possible to identify whether the molecules tested can potentially exhibit valuable secondary activity in addition to their UV filter and antioxidant properties. Kojic acid can be used as a reference in terms of in tubo tyrosinase inhibition [41]. In order to position our molecules with respect to this reference, we determined the EC_50_ value, defined as the concentration (in μM) of product needed to reduce 50% of the tyrosinase activity (Table 2). The lower the EC_50_ value, the better the inhibition.

Interesting values are obtained for the phenolic series, in particular for compounds **1**, **2**, and **4** that have EC_50_ very close to that of kojic acid. Some molecules of the furan series exhibit EC_50_ which is relatively high (>100 µM) or has no activity at all. Pyrrolic series present no activity. Through this preliminary study of tyrosinase inhibition, three molecules were found competitive against kojic acid, but these results need to be confirmed on cells.

### 2.5. UV Filter Combinations

As highlighted by the three types of analyses conducted above, some compounds exhibit remarkable properties and can cover a wide range of wavelengths in the UV-A/blue light regions. Organic UV filters are usually used in mixtures to afford a wide coverage of wavelengths [42]. In a similar fashion, we combined specific compounds to study the properties of the resulting mixtures. One molecule of each series was chosen: compound **4** from the phenolic series for its antiradical and tyrosinase inhibition activities; compound **8** from the furanic series for its proximity with avobenzone UV spectra and stability; and compound **13** from the pyrrolic series for its blue light coverage and high absorbance. These three compounds allowed the obtention of four different mixtures: **A** (compounds **4** + **13**), **B** (compounds **4** + **8**), **C** (compounds **8** + **13**), and **D** (compounds **4** + **8** + **13**), which were submitted to the same tests conducted on the individual compounds (i.e., UV properties, loss of absorbance, antiradical activity, and tyrosinase inhibition).

**Figure 3:**
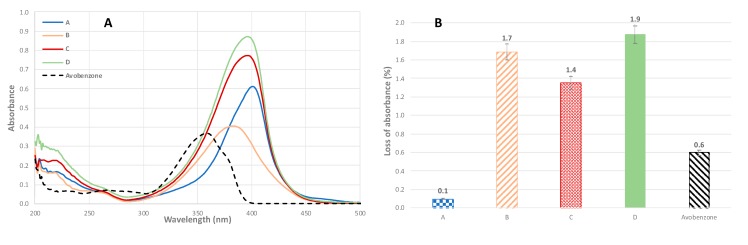
UV spectra (**A**) and loss of absorbance (**B**) for mixtures **A**, **B**, **C**, and **D** (individual concentration of compounds 10 μmol.L^−1^ in ethanol).

Spectra obtained for the mixtures are presented in Figure 3. As can be expected when mixing compounds showing similar UV profiles, combination of the molecules led to increased absorbance and wider wavelength coverage, allowing protection in the entire UV-A and part of the blue light regions. In addition, loss of absorbance upon UV irradiation was decreased. When looking at mixture **A**, for example, UV radiations led only to a very small loss of absorbance (0.1%) while individual molecules **4** and **13** showed more degradation (6.4% and 3.4% loss of absorbance, respectively). This could imply a synergic effect between the molecules. All three other mixtures exhibited the same behavior, leading to higher photostability than their individual components. Only mixture **A** (0.1%) resulted in better photostability than the reference avobenzone (0.6%), while mixtures **B**, **C**, and **D** showed very interesting photostability with 1.7%, 1.4% and 1.9% loss of absorbance, respectively. Combining the compounds resulted in an impressive impact on the stability toward UV radiations, while conserving the other properties. Indeed, as shown in Table 3, values of EC_50_ (DPPH and tyrosinase inhibition) obtained for the mixtures were in the same order of magnitude as the individual compounds.

### 2.6. Endocrine Disruption Assays

**Figure 4:**
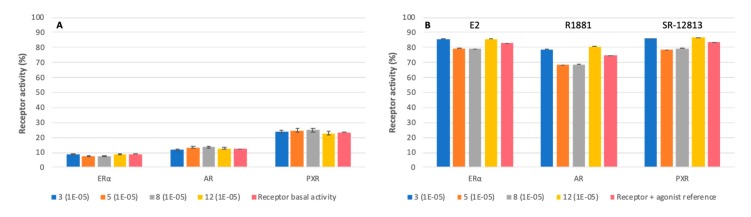
Receptor activities (%) of estrogen receptor α (ERα), androgen receptor (AR), and pregnane X receptor (PXR) of synthesized molecules for agonist (**A**) and antagonist (**B**) interactions at 10^−5^ mol.L^−1^.

In the last years, studies have shed light on the ability of organic UV filters to act as endocrine disruptors [43,44] whose continuous exposure can cause serious developmental effects on reproductive organs and the central nervous system [45]. To evaluate potential health risks associated to the newly synthesized UV filters, their ability to interact with estrogen receptor α (ERα), androgen receptor (AR), and pregnane X receptor (PXR) was evaluated. ERα activity is regulated by the steroid estrogen sex hormone E2 (17β-estradiol). AR is activated by binding with any androgenic hormones, and finally PXR is a member of the steroid and xenobiotic sensing nuclear receptors family. All three receptors were tested in the absence (agonist test) or presence (antagonist test) of their respective agonist control molecule, E2 (17β-estradiol), R1881 (methyltrienolone), and SR-12813 for ERα, AR, and PXR, respectively. Endocrine disruption assays were performed on four molecules—**3**, **5**, **8**, and **12**—as they present the basic structures for phenolic, furfural, 5-hydroxymethylfurfural (HMF), and pyrrole derivatives. Tests were performed using different concentrations (from 3 × 10^−7^ to 3 × 10^−5^ M) and we focused on the results for the concentration of synthesized molecules needed to obtain the previously described UV properties (i.e., 1 × 10^−5^ M). Results in Figure 4A show that the four molecules barely interact with receptors, demonstrating the absence of agonist effects. For antagonist properties, synthesized molecules were mixed with the agonist control molecule of each receptor. As shown in Figure 4B, receptors conserved their specific interactions toward the agonist reference even in the presence of the synthesized compounds, confirming the absence of antagonistic activities. These preliminary results demonstrated the innocuousness of these compounds, pushing to seriously consider them as a replacement for current petroleum-based UV filters.

## 3. Conclusions

Herein, the UV properties, photostability, antiradical and tyrosinase inhibition properties of bio-based organic UV filters deriving from Meldrum’s acid are described. Study of the structure–activity relationships showed that UV properties of such molecules can be fine-tuned by judiciously playing with the level of conjugation and congestion on the aromatic moiety. Moreover, some of the compounds evaluated in this study proved to possess competitive secondary activities such as antiradical and tyrosinase inhibitory properties. Mixtures of compounds from the different series provided higher UV properties competitive with the commercial reference (i.e., avobenzone) while retaining the secondary activities of the individual molecules. Such mixtures can then be perfectly adapted according to the type of UV protection desired but also to the secondary activity sought for the formulation. Finally, endocrine disruption assays revealed the innocuousness of the synthesized compounds, making them promising substitutes for current, toxic fossil-based organic UV filters such as avobenzone.

## 4. Materials and Methods

Syringaldehyde, vanillin, 4-hydroxybenzaldehyde, 3,4-dihydroxybenzaldehyde, 3,4,5-trimethoxybenzaldehyde, furfural, 5-(hydroxymethyl)furfural, 4,5-dimethyl-2-furaldehyde, 2,5-furandicarboxaldehyde, 5-ethyl-2-furaldehyde, tyrosinase from mushroom, and 2,2-di(4-tert-octylphenyl)-1-picrylhydrazyl (DPPH) were purchased from Sigma Aldrich (St. Louis, MO, USA). Meldrum’s acid, pyrrole-2-carboxaldehyde, *N*-methyl-2-pyrrolecarboxaldehyde, and indole-2-carboxaldehyde were purchased from TCI (Tokyo, Japan). 5-Acetoxymethyl-2-furanaldehyde, 2-benzofurancarboxaldehyde, and all solvents were purchased from Fischer Scientific (Hampton, NH, USA). All chemicals were used directly without purification.

^1^H-NMR spectra were recorded on a Brucker Fourier 300 (300 MHz) (Billerica, MA, USA) and were calibrated with residual acetone-*d*_6_, DMSO-*d*_6_, or CDCl_3_ proton signals at δ 2.05, 2.50, or 7.26 ppm, respectively. Data are reported as follows: chemical shift (δ ppm), multiplicity (s = singlet, d = doublet, t = triplet, q = quartet, dd = doublet of doublets, dt = doublet of triplets, ddd = doublet of doublets of doublets, dddd = doublet of doublets of doublets of doublets, and m = multiplet), coupling constant (Hz), integration, and assignment. ^13^C-NMR spectra were recorded on a Brucker Fourier 300 (75 MHz) (Billerica, MA, USA) and were calibrated with acetone-*d*_6_, DMSO-*d*_6_, or CDCl_3_ signals at δ 29.84, 39.52, or 77.16 ppm, respectively. Data are reported as follows: chemical shift (δ ppm) and attribution. All NMR assignments were made using COSY, HMBC, and HSQC spectrum. Melting points were recorded on a Mettler Toledo MP50 Melting Point System (Greifensee, Switzerland) (heating at 4 °C/min). High resolution mass spectrometry was performed on an Agilent 1290 system, equipped with a PDA UV detector, and a 6545 Q-TOF mass spectrometer (Wilmington, DE, USA). The source was equipped with a JetStream ESI probe operating at atmospheric pressure.

### 4.1. General Synthesis Procedure

A mixture of Meldrum’s acid (6.9 mmol, 1 equiv, 1 g) and the corresponding conjugated aldehyde (6.9 mmol, 1 equiv) in deionized water (10 mL) was stirred at 75 °C for 2–4 h. Then, the precipitate was filtered-off and dried to afford the targeted compounds. In case of no precipitate, the reaction mixture was extracted with ethyl acetate and the combined organic layers were dried over anhydrous magnesium sulfate, filtered, and dried in vacuo.

*5-[(4-Hydroxy-3,5-dimethoxyphenyl)methyl]-2,2-dimethyl-1,3-dioxane-4,6-dione* (**1**): filtration led to a yellow powder (87%). m.p. 164–166 °C, UV: λ_max_ (EtOH, nm) 405, ε (L mol^−1^.cm^−1^) 24614. ^1^H-NMR (300 MHz, CDCl_3_) δ: 8.33 (s, 1H, H3), 7.73 (s, 2H, H5–5′), 6.28 (s, 1H, OH), 3.97 (s, 6H, H8–8′), 1.79 (s, 6H, H10–10′). ^13^C-NMR (75 MHz, CDCl_3_) δ: 164.4 (C1 or C1′), 160.8 (C1 or C1′), 158.8 (C3), 146.8 (C6–6′), 141.6 (C7), 123.6 (C4), 113.0 (C5–5′), 110.8 (C2), 104.4 (C9), 56.6 (C8–8′), 27.6 (C10–10′). HRMS: *m/z* [M + Na]^+^ calculated for C_15_H_16_O_7_Na: 331.0794 found: 331.0793 

*5-(4-Hydroxy-3-methoxybenzylidene)-2,2-dimethyl-1,3-dioxane-4,6-dione* (**2**): filtration led to a yellow powder (87%). m.p. 124–126 °C, UV: λ_max_ (EtOH, nm) 393, ε (L mol^−1^.cm^−1^) 29453. ^1^H-NMR (300 MHz, DMSO-*d*_6_) δ: 10.71 (s, 1H, OH), 8.26 (s, 1H, H3), 8.12 (d, *J* = 2.01 Hz, 1H, H9), 7.78 (dd, *J* = 2.01 Hz ; 8.58 Hz, 1H, H5), 6.92 (d, *J* = 8.4 Hz, 1H, H6), 3.81 (s, 3H, H10), 1.72 (s, 6H, H12–12′). ^13^C-NMR (75 MHz, DMSO-d6) δ: 164.0 (C1 or C1′), 160.8 (C1 or C1′), 158.0 (C3), 154.2 (C7), 147.7 (C8), 132.5 (C5), 123.9 (C4), 118.0 (C9), 116.0 (C6), 110.1 (C2), 104.3 (C11), 56.1 (C10), 27.3 (C12–12′). HRMS: *m/z* [M + Na]^+^ calculated for C_14_H_14_O_6_Na: 301.0688 found: 301.0686 

*5-(4-Hydroxybenzylidene)-2,2-dimethyl-1,3-dioxane-4,6-dione* (**3**): filtration led to a yellow powder (90%). m.p. 198–200 °C, UV: λ_max_ (EtOH, nm) 374, ε (L mol^−1^.cm^−1^) 29413. ^1^H-NMR (300 MHz, DMSO-*d*_6_) δ: 8.25 (s, 1H, H3), 8.17 (d, *J* = 8.8 Hz, 2H, H5–5′), 6.90 (d, *J* = 8.8 Hz, 2H, H6–6′), 1.72 (s, 6H, H9–9′). ^13^C-NMR (75 MHz, DMSO-*d*_6_) δ: 163.7 (C7), 163.4 (C1 or C1′), 160.3 (C1 or C1′), 157.1 (C3), 138.0 (C5–5′), 123.1 (C4), 115.9 (C6–6′), 109.9 (C2), 104.0 (C8), 26.9 (C9–9′). HRMS: *m/z* [M + Na]^+^ calculated for C_13_H_12_O_5_Na: 271.0582 found: 271.0582 

*5-(3,4-Dihydroxybenzylidene)-2,2-dimethyl-1,3-dioxane-4,6-dione* (**4**): filtration led to a yellow powder (88%). m.p. 155–156 °C, UV: λ_max_ (EtOH, nm) 398, ε (L mol^−1^.cm^−1^) 26615. ^1^H-NMR (300 MHz, DMSO-*d*_6_) δ: 10.52 (s, 1H, OH), 9.57 (s, 1H, OH), 8.14 (s, 1H, H3), 7.92 (d, *J* = 2.4 Hz, 1H, H9), 7.54 (dd, *J* = 2.1, 8.7 Hz, 2H, H5–5′), 6.87 (d, *J* = 8.1 Hz, 1H, H6), 1.78 (s, 6H, H11–11′). ^13^C-NMR (75 MHz, DMSO-*d*_6_) δ: 163.5 (C1 or C1′), 160.2 (C1 or C1′), 157.4 (C3), 153.1 (C7), 145.2 (C8), 131.3 (C5), 123.5 (C4), 120.3 (C9), 115.6 (C6), 109.4 (C2), 103.9 (C10), 26.8 (C11–11′). HRMS: *m/z* [M + Na]^+^ calculated for C_13_H_12_O_6_Na: 287.0532 found: 287.0522 

*5-(2-furanylmethylene)-2,2-dimethyl-1,3-dioxane-4,6-dione* (**5**): filtration led to brownish powder (85%). m.p. 82–84 °C, UV: λ_max_ (EtOH, nm) 361, ε (L mol^−1^.cm^−1^) 33809. ^1^H-NMR (300 MHz, DMSO-*d*_6_) δ: 8.33 (d, *J* = 1.2 Hz, 1H, H7), 8.26 (d, *J* = 3.8 Hz, 1H, H5), 8.12 (s, 1H, H3), 6.96 (ddd, *J* = 0.6, 1.6, 3.8 Hz, 1H, H6), 1.71 (s, 6H, H9–9′). ^13^C-NMR (75 MHz, DMSO-d6) δ: 162.7 (C1 or 1′), 159.8 (C1 or 1′), 152.1 (C7), 149.4 (C4), 139.6 (C3), 127.8 (C5), 115.6 (C6), 107.9 (C2), 104.6 (C8), 27.0 (C9–9′). HRMS: *m/z* [M + Na]^+^ calculated for C_11_H_10_O_5_Na: 245.0426 found: 245.0431

*5-[(4,5-dimethyl-2-furanyl)methylene]-2,2-dimethyl-1,3-dioxane-4,6-dione* (**6**): filtration led to brown powder (90%). m.p. 127–129 °C, UV: λ_max_ (EtOH, nm) 400, ε (L mol^−1^.cm^−1^) 37961. ^1^H-NMR (300 MHz, DMSO-d6) δ: 8.22 (s, 1H, H5), 7.98 (s, 1H, H3), 2.40 (s, 3H, H8), 2.05 (s, 3H, H9), 1.69 (s, 6H, H11–11′). ^13^C-NMR (75 MHz, DMSO-d6) δ: 163.4 (C1 or 1′), 161.1 (C1 or 1′), 160.5 (C7), 147.8 (C4), 139.1 (C3), 132.3 (C5), 122.7 (C2), 104.6 (C10), 27.3 (C11–11′), 12.8 (C8), 9.9 (C9). HRMS: *m/z* [M + Na]^+^ calculated for C_13_H_14_O_5_Na: 273.0739 found: 273.0744 

*5-[(5-ethyl-2-furanyl)methylene]-2,2-dimethyl-1,3-dioxane-4,6-dione* (**7**): filtration led to brown powder (87%). m.p. 50–52 °C, UV: λ_max_ (EtOH, nm) 374, ε (L mol^−1^.cm^−1^) 31307. ^1^H-NMR (300 MHz, DMSO-*d*_6_) δ: 8.29 (d, *J* = 3.8 Hz, 1H, H5), 8.06 (s, 1H, H3), 6.71 (m, 1H, H6), 2.82 (q, *J* = 7.6 Hz, 2H, H8), 1.70 (s, 6H, H11–11′), 1.25 (t, *J* = 7.6 Hz, 3H, H9). ^13^C-NMR (75 MHz, DMSO-*d*_6_) δ: 168.2 (C1 or 1′), 162.9 (C1 or 1′), 159.9 (C7), 148.4 (C4), 139.0 (C3), 130.3 (C5), 111.9 (C6), 105.4 (C2), 104.2 (C10), 26.9 (C8), 21.5 (C11–11′), 11.4 (C9). HRMS: *m/z* [M + Na]^+^ calculated for C_13_H_14_O_5_Na: 273.0739 found: 273.0734 

*5-[[5-(hydroxymethyl)-2-furanyl]methylene]-2,2-dimethyl-1,3-dioxane-4,6-dione* (**8**): filtration led to brown light solid (82%). m.p. 92–94 °C, UV: λ_max_ (EtOH, nm) 374, ε (L mol^−1^.cm^−1^) 28814. ^1^H-NMR (300 MHz, DMSO-*d*_6_) δ: 8.29 (d, *J* = 3.8 Hz, 1H, H5), 8.07 (s, 1H, H3), 6.80 (d, 1H, *J* = 3.8 Hz, H6), 5.67 (s, 1H, OH), 4.58 (s, 2H, H8), 1.71 (s, 6H, H10–10′). ^13^C-NMR (75 MHz, DMSO-*d*_6_) δ: 165.6 (C1 or 1′), 162.7 (C1 or 1′), 159.8 (C7), 148.7 (C4), 139.3 (C3), 129.3 (C5), 112.6 (C6), 106.6 (C2), 104.4 (C9), 56.3 (C8), 26.9 (C10–10′). HRMS: *m/z* [M + Na]^+^ calculated for C_12_H_12_O_6_Na: 275.0532 found: 275.0535 

*5-(2-benzofuranylmethylene)-2,2-dimethyl-1,3-dioxane-4,6-dione* (**9**): filtration led to yellow powder (78%). m.p. 122–124 °C, UV: λ_max_ (EtOH, nm) 386, ε (L mol^−1^.cm^−1^) 32310. ^1^H-NMR (300 MHz, DMSO-*d*_6_) δ: 8.57 (s, 1H, H5), 8.22 (s, 1H, H3), 7.93 (d, *J* = 7.8 Hz, 1H, H7), 7.72 (d, *J* = 8.4 Hz, 1H, H10), 7.60 (m, 1H, H8), 7.40 (t, *J* = 7.5 Hz, 1H, H9), 1.75 (s, 6H, H13–13′). ^13^C-NMR (75 MHz, DMSO-*d*_6_) δ: 162.3 (C1 or 1′), 159.3 (C1 or 1′), 156.2 (C11), 149.7 (C4), 139.9 (C3), 129.9 (C8), 127.9 (C6), 124.4 (C9), 124.0 (C7), 122.0 (C5), 112.5 (C10), 112.0 (C2), 104.9 (C12), 27.1 (C13–13′). HRMS: *m/z* [M + Na]^+^ calculated for C_15_H_12_O_5_Na: 295.0582 found: 295.0577 

*5-[[5-[(acetyloxymethyl)-2-furanyl]methylene]-2,2-dimethyl-1,3-dioxane-4,6-dione* (**10**): extraction led to brown oil (86%). UV: λ_max_ (EtOH, nm) 366, ε (L mol^−1^.cm^−1^) 28728. ^1^H-NMR (300 MHz, DMSO-*d*_6_) δ: 8.23 (m, 1H, H5), 8.06 (m, 1H, H3), 6.93 (m,1H, H6), 5.19 (m, 2H, H8), 2.09 (m, 3H, H10), 1.72 (m, 6H, H12–12′). ^13^C-NMR (75 MHz, DMSO-*d*_6_) δ: 169.9 (C9), 162.5 (C1 or 1′), 159.6 (C1 or 1′), 158.0 (C7), 149.3 (C4), 139.1 (C3), 128.1 (C5), 115.0 (C6), 108.4 (C2), 104.6 (C11), 57.5 (C8), 27.0 (C12), 20.5 (C10). HRMS: *m/z* [M + Na]^+^ calculated for C_14_H_14_O_7_Na: 317.0637 found: 317.0637 

*5,5′-(2,5-furandiyldimethylidene)bis [2,2-dimethyl-1,3-dioxane-4,6-dione]* (**11**): filtration led to yellow powder (75%). m.p. 204–206 °C, UV: λ_max_ (EtOH, nm) 406, 426, ε (L mol^−1^.cm^−1^) 47642. ^1^H-NMR (300 MHz, DMSO-*d*_6_) δ: 8.22 (s, 2H, H3), 8.06 (s, 2H, H5), 1.74 (s, 6H, H7–7′). ^13^C-NMR (75 MHz, DMSO-*d*_6_) δ: 162.1 (C1 or 1′), 159.2 (C1 or 1′), 152.9 (C4), 137.5 (C5), 127.4 (C3), 113.9 (C2), 105.1 (C6), 27.2 (C7–7′). HRMS: *m/z* [M + H]^+^ calculated for C_18_H_16_O_9_: 377.0873 found: 377.0874 

*5-(1H-pyrrol-2-ylmethylidyne)-2,2-dimethyl-1,3-dioxane-4,6-dione* (**12**): filtration led to a green powder (90%). m.p. 178–180 °C, UV: λ_max_ (EtOH, nm) 393, ε (L mol^−1^.cm^−1^) 40225. ^1^H-NMR (300 MHz, Acetone-*d*_6_) δ: 8.21 (s, 1H, H3), 7.72 (dddd, *J* = 0.9, 1.5, 2.4, 3.3 Hz, 1H, H6), 7.35 (s, 1H, H5), 6.59 (dt, *J* = 2.2, 4.2, Hz, 1H, H7), 1.73 (s, 6H, H9–9′). ^13^C-NMR (75 MHz, DMSO-*d*_6_) δ: 164.7 (C1 or 1′), 164.2 (C1 or 1′), 143.5 (C3), 133.8 (C6), 131.1 (C5), 129.5 (C4), 114.8 (C7), 104.7 (C8), 27.2 (C9–9′). HRMS: *m/z* [M + Na]^+^ calculated for C_11_H_11_O_4_NNa: 244.0586 found: 244.0580 

*5-(1-methyl-1H-pyrrol-2-ylmethylidene)2,2-dimethyl-1,3-dioxane-4,6-dione* (**13**): filtration led to brown powder (72%). m.p. 102–104°C, UV: λ_max_ (EtOH, nm) 401, ε (L mol^−1^.cm^−1^) 43003. ^1^H-NMR (300 MHz, DMSO-*d*_6_) δ: 8.34 (m, 1H, H5), 8.29 (s, 1H, H3), 7.53 (t, *J* = 2.0 Hz, 1H, H6), 6.43 (ddd, *J* = 0.8, 2.4, 4.5 Hz, 1H, H7), 3.98 (s, 3H, H8), 1.70 (s, 6H, H10–10′). ^13^C-NMR (75 MHz, DMSO-*d*_6_) δ: 165.0 (C1 or 1′), 161.3 (C1 or 1′), 140.4 (C3), 137.0 (C6), 129.8 (C4), 128.0 (C5), 112.9 (C7), 104.0 (C9), 103.1 (C2), 34.9 (C8), 27.3 (C10). HRMS: *m/z* [M + Na]^+^ calculated for C_12_H_13_O_4_NNa: 258.0742 found: 258.0738 

*5-(1H-indol-2-ylmethylene)-2,2-dimethyl-1,3-dioxane-4,6-dione* (**14**): filtration led to a yellow powder (57%). m.p. 140–142 °C, UV: λ_max_ (EtOH, nm) 416, ε (L mol^−1^.cm^−1^) 39433. ^1^H-NMR (300 MHz, DMSO-*d*_6_) δ: 11.78 (s, 1H, NH), 8.42 (s, 1H, H3), 7.84 (s, 1H, H5), 7.73 (t, 2H, *J* = 7.4 Hz, H8–9), 7.40 (t, 1H, *J* = 7.7 Hz, H7), 7.14 (t, 1H, *J* = 7.5 Hz, H10), 1.75 (s, 6H, H13–13′). ^13^C-NMR (75 MHz, DMSO-*d*_6_) δ: 163.0 (C1–1′), 161.9 (C1–1′), 144.3 (C3), 140.4 (C6), 131.4 (C8), 128.3 (C7), 127.4 (C11), 122.9 (C4), 122.3 (C5), 121.4 (C10), 113.6 (C9), 106.8 (C2), 104.5 (C12), 26.7 (C13–13′). HRMS: *m/z* [M + Na]^+^ calculated for C_15_H_13_O_4_NNa: 294.0742 found: 294.0738 

### 4.2. UV Analysis and Loss of Absorbance

UV–Vis spectra were recorded on a Cary 60 UV–Vis by Agilent (Wilmington, DE, USA) from a solution of the desired compound at 10 µmol.L^−1^ in ethanol placed in a 1 cm quartz cuvette and are reported in wavelength (nm). Loss of absorbance was obtained by irradiating the solutions for 1 h into a Rayonet^®^ RPR-200 (λ = 300 nm, P = 8.32 W/m^2^, stirring, T = 35 °C) from SNE Ultraviolet Co (Branford, CT, USA) using 14 RPR-3000A lamps. Absorbance after 1 h was compared to the one before irradiation and loss of absorbance was calculated in percentage at the λ_max_.

### 4.3. DPPH Inhibition

Antiradical activities were determined via 2,2-diphenyl-1-picrylhydrazyl (DPPH) assay to determine EC_50_ values. The test started with the addition of 190 μL of DPPH solution (C = 200 μM) in ethanol to a well containing 10 μL of potential antiradical solution in ethanol (concentrations from 400 to 12.5 μM) [46]. Reaction was carried out in a Multiskan FC system from Thermo Fisher Scientific (Waltham, MA, USA) and disappearance of the DPPH radicals was monitored at 520 nm every 5 min for 7.5 h. Use of different concentrations of potential antiradical gave the EC_50_ value which is the quantity needed to reduce half the initial population of DPPH radicals.

### 4.4. Tyrosinase Inhibition

Tyrosinase inhibitor activity was measured by spectrophotometry based on the method presented by Masamoto et al. [47]. First, 10 μL of inhibitor solution at different concentrations (from 5000 to 0.1 μM) in DMSO were added to a 96-well microplate and mixed with ammonium formate buffer (60 μL, 50 mmol.L^−1^, pH 6.4). Then, 20 μL (0.8 mg.mL^−1^) of tyrosine in ammonium formate buffer were added. Finally, 10 μL of mushroom tyrosinase (5000 U.mL^−1^ in ammonium formate buffer) were added and the assay mixture was then incubated at 37 °C for 10 min. After incubation, the amount of dopachrome production in the reaction mixture was determined by analysis at 450 nm in a microplate spectrophotometer. Kojic acid solution at different concentrations was used as a positive control. The concentration needed for 50% of tyrosinase inhibition (IC_50_) was determined.

### 4.5. Endocrine Disruption Assays

The agonistic and antagonistic potentials of Meldrum’s acid derivatives were analyzed following a literature method in which ERα, PXR, and AR transcriptional activities were monitored by using corresponding reporter cells HELN ERα, HELN AR, and HG5LN PXR cells, respectively [48]. Activities were measured in relative light units (RLUs) and 100% of activities were assigned to the RLU value obtained with 10 nM agonist control (estradiol (E2), R 1881, SR 12813). The sample (DMSO) was tested as a control without any compound.

## 5. Patents

F.A., C.P., and M.M.M. filed a patent based on the work described here (Patent application no. FR1912971).

## Figures and Tables

**Table 1:** EC_50_ of synthesized molecules for DPPH (2,2-diphenyl-1-picrylhydrazyl) inhibition in ethanol.

Series/References	Compound	EC_50_ (nmol)	EC_50_ (mM)
Phenolic series	**1**	3.94 ± 0.20	19.70 ± 1.00
	**2**	>80	>400
	**3**	>80	>400
	**4**	3.34 ± 0.17	16.70 ± 0.80
Furanic series	**5**	>80	>400
	**6**	- ^a^	- ^a^
	**7**	- ^a^	- ^a^
	**8**	>80	>400
	**9**	- ^a^	- ^a^
	**10**	- ^a^	- ^a^
	**11**	>80	>400
Pyrrolic series	**12**	- ^a^	- ^a^
	**13**	- ^a^	- ^a^
	**14**	- ^a^	- ^a^
References	**BHA**	5.74 ± 0.29	28.70 ± 1.45
	**BHT**	7.61 ± 0.38	38.05 ± 1.9

^a^ No activity observed.

**Table 2:** EC_50_ of synthesized molecules for mushroom tyrosinase inhibition.

Series/Reference	Compound	EC_50_ (µM)
Phenolic series	**1**	17 ± 2
	**2**	23 ± 3
	**3**	106 ± 13
	**4**	13 ± 2
Furanic series	**5**	465 ± 56
	**6**	- ^a^
	**7**	- ^a^
	**8**	- ^a^
	**9**	252 ± 30
	**10**	- ^a^
	**11**	- ^a^
Pyrrolic series	**12**	- ^a^
	**13**	- ^a^
	**14**	- ^a^
Reference	**Kojic Acid**	18 ± 2

^a^ No activity observed.

**Table 3:** EC_50_ of mixtures for DPPH and tyrosinase inhibition.

Mixture	DPPH Inhibition EC_50_ (nmol)	DPPH Inhibition EC_50_ (μM)	Tyrosinase Inhibition EC_50_ (μM)
**A**	3.17 ± 0.16	15.85 ± 0.80	14 ± 2
**B**	3.12 ± 0.16	15.60 ± 0.80	13 ± 2
**C**	>80	>400	- ^a^
**D**	3.15 ± 0.16	15.75 ± 0.80	24 ± 3

^a^ No activity observed.

## Supplementary Materials

The following are available online: the ^1^H and ^13^C-NMR spectra, UV spectrum, loss of absorbance study, DPPH assay, and tyrosinase inhibitor assay are provided for every single molecule synthesized in this work.
