# Supplementary material for: Innovative Bio-Based Organic UV-A and Blue Light Filters from Meldrum’s Acid
Source: Molecules. 2020 May 6;25(9):2178. doi: 10.3390/molecules25092178 (PMC7248827; doi:10.3390/molecules25092178)
Supplement: Supplementary file 1 [file molecules-25-02178-s001.pdf]

Supplementary Information

Innovative bio-based organic UVA & blue light filters from Meldrum's acid

Cédric Peyrot [1], Matthieu M. Mention [1], Fanny Brunissen [1], Patrick Balaguer [2] and Florent Allais [1,*]

[1] URD Agro-Biotechnologies Industrielles (ABI), CEBB, AgroParisTech, 51110 Pomacle, France; cedric.peyrot@agroparistech.fr (C.P.); matthieu.mention@agroparistech.fr (M.M.M.); fanny.brunissen@agroparistech.fr (F.B.)

[2] IRCM, Inserm, Univ Montpellier, ICM, 208 Avenue des Apothicaires 34298 Montpellier Cedex 5, France; patrick.balaguer@icm.unicancer.fr

* Correspondence: florent.allais@agroparistech.fr;

Commented [M1]: Please carefully check the accuracy of names and affiliations. Changes will not be possible after proofreading.

Commented [M2]: In system it is Matthieu Mention, please confirm which is correct

Table of Contents

1. ^{1}H & ^{13}C NMR spectra 2
2. UV Spectra 25
   2.1. Phenolic series 25
   2.2. Furanic series 26
   2.3. Pyrrolic series 28
   2.4. Mixtures 29
   2.5. Reference 30
3. Loss of absorbance (LoA) 31
   3.1. Phenolic series 31
   3.2. Furanic series 33
   3.3. Pyrrolic series 36
   3.4. Mixtures 38
   3.5. Reference 40
4. DPPH assays 41
   4.1. Phenolic series 41
   4.2. Furanic series 42
   4.3. Pyrrolic series 44
   4.4. Mixtures 45
   4.5. References 46
5. Tyrosinase inhibition 47

1. **H & ^{13}C NMR spectra**

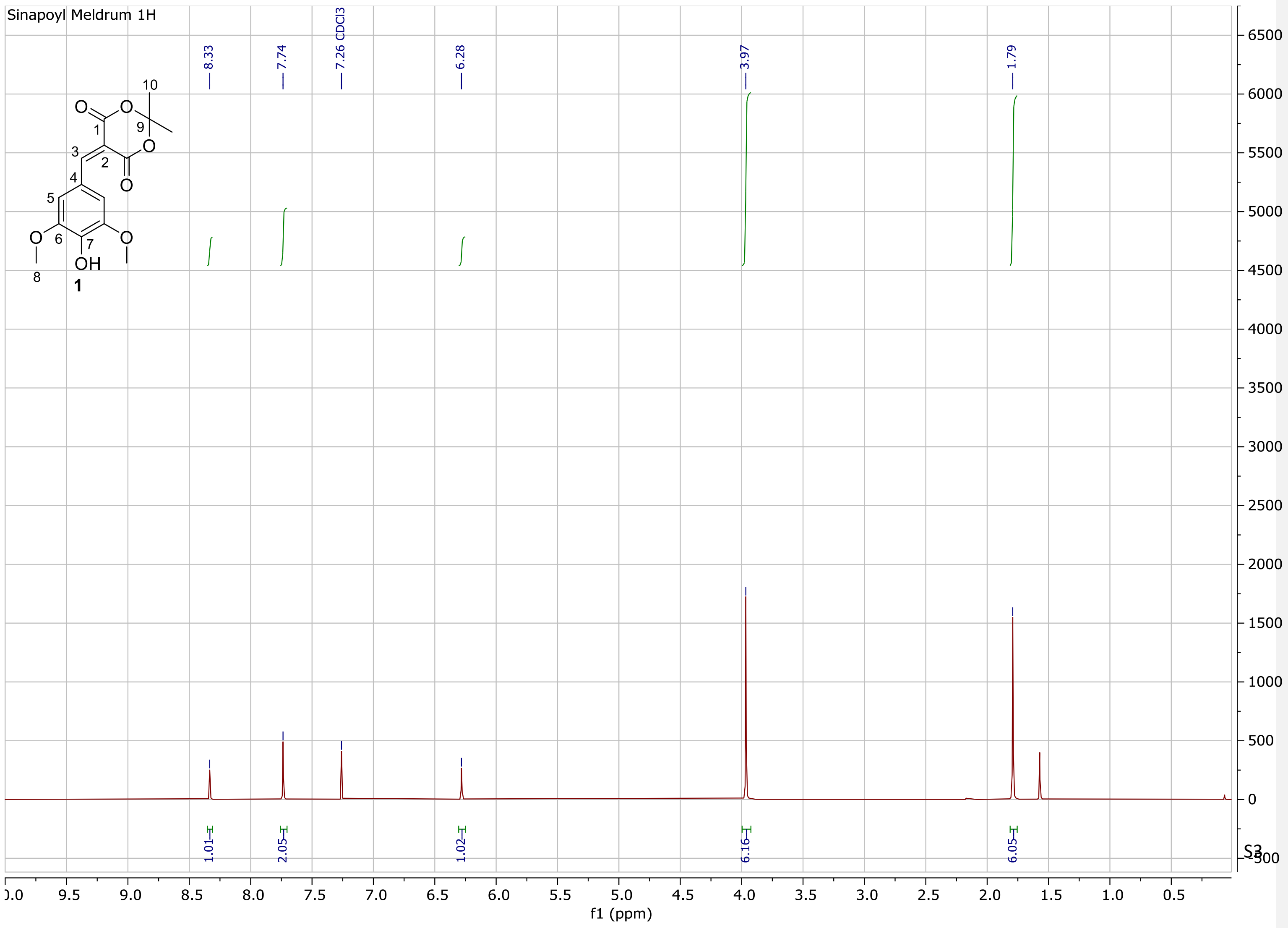
Sinapoyl Meldrum 1H
8.33
7.74
7.26 CDCl3
6.28
3.97
1.79
1.01
2.05
1.02
6.16
6.05
1
f1 (ppm)

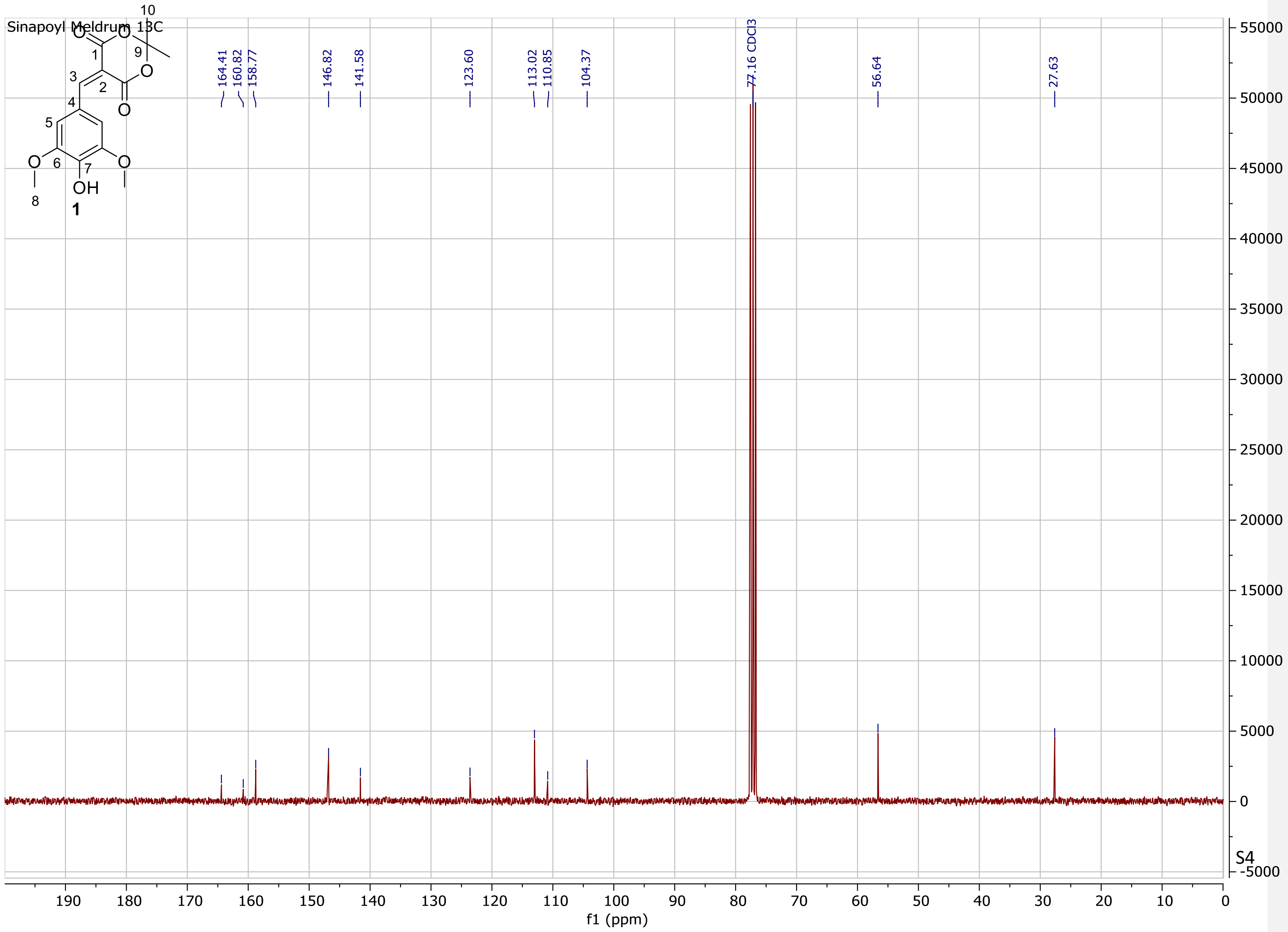
Sinapoyl Meldrum 13C
164.41
160.82
158.77
146.82
141.58
123.60
113.02
110.85
104.37
77.16 CDCl3
56.64
27.63
f1 (ppm)

S4

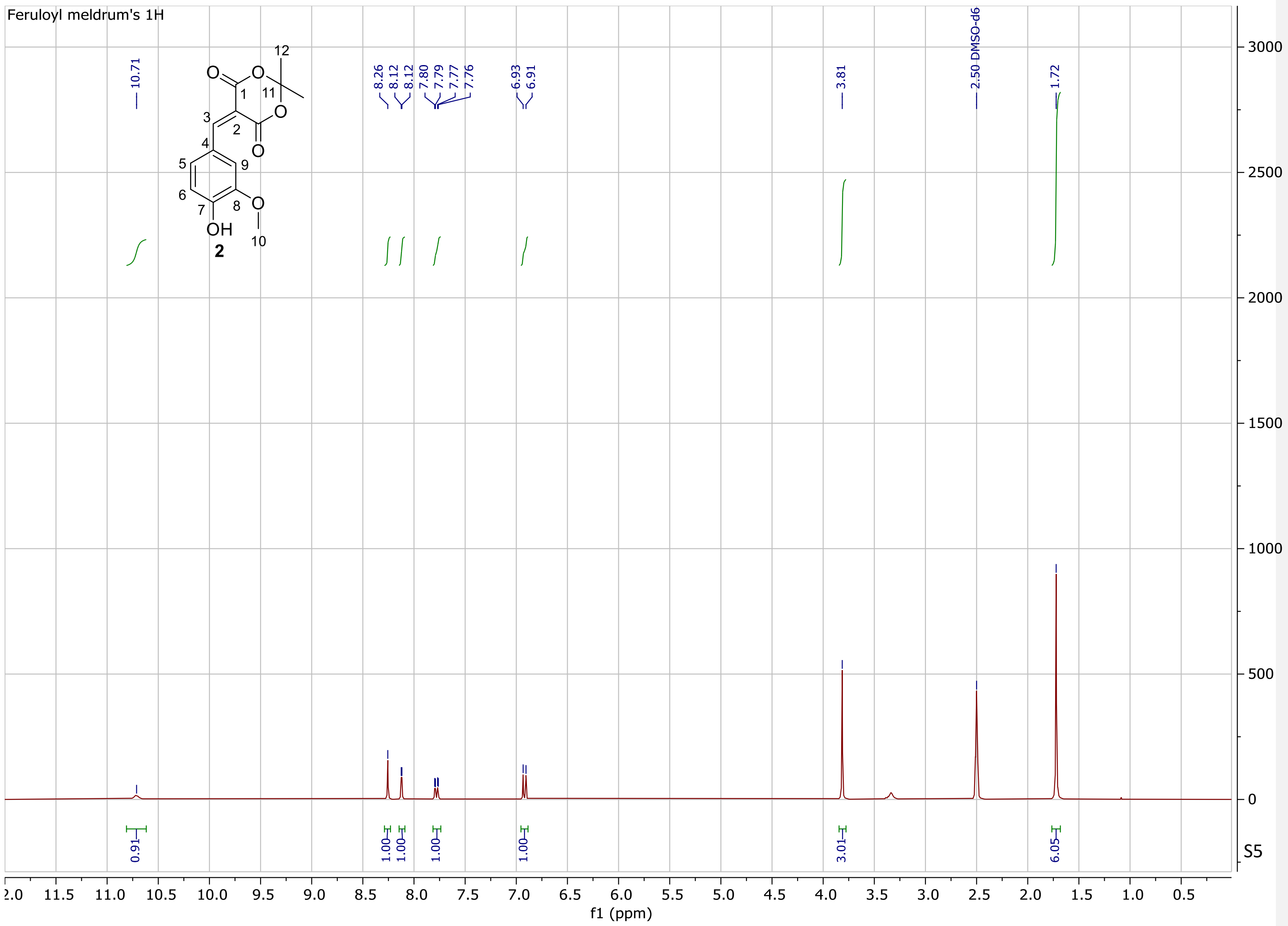
Feruloyl meldrum's 1H
10.71
8.26
8.12
8.12
7.80
7.79
7.77
7.76
6.93
6.91
3.81
2.50 DMSO-d6
1.72
0.91
1.00
1.00
1.00
1.00
3.01
6.05
2
f1 (ppm)

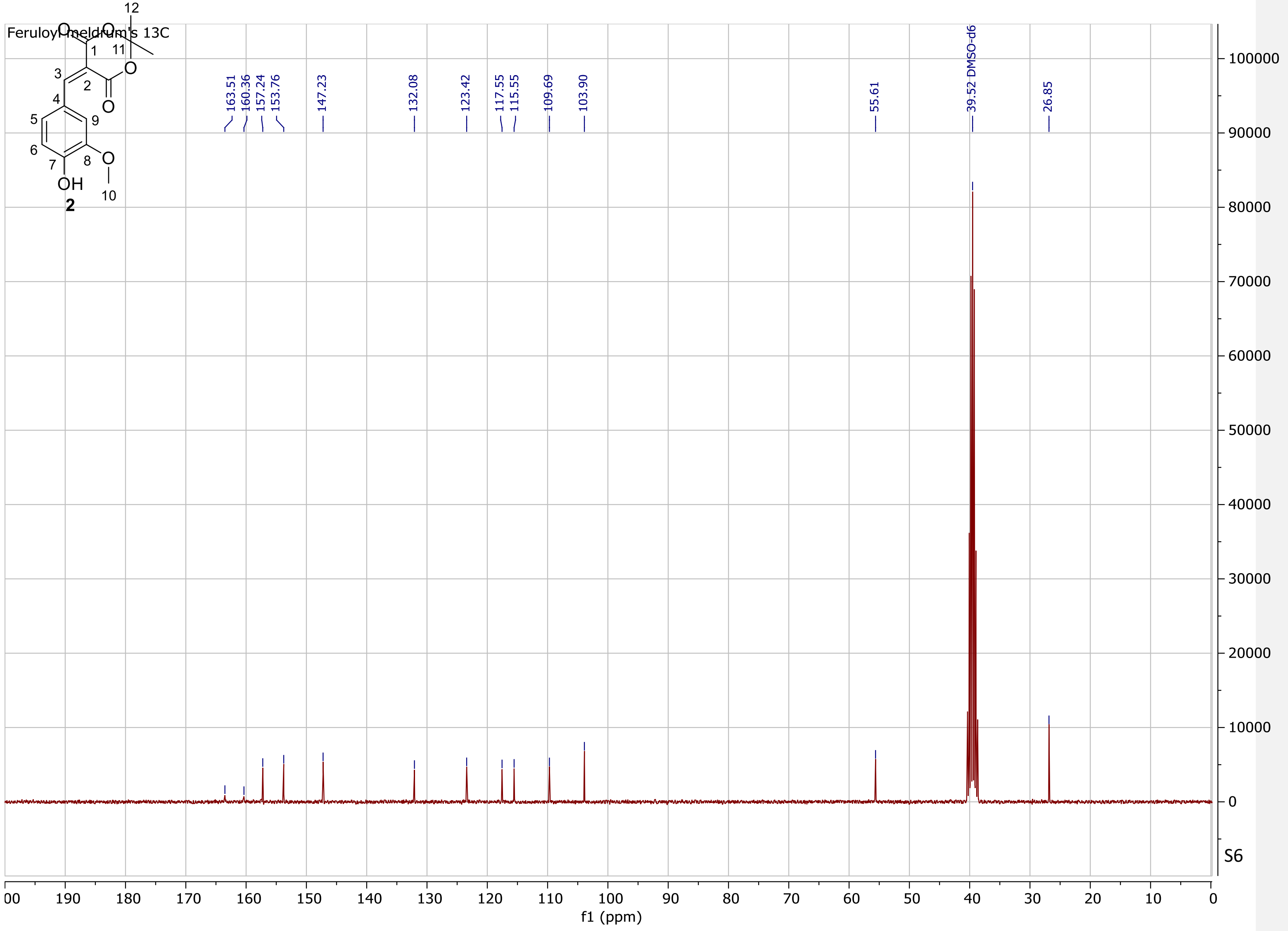
Feruloyl-meldrum's 13C
163.51
160.36
157.24
153.76
147.23
132.08
123.42
117.55
115.55
109.69
103.90
55.61
39.52 DMSO-d6
26.85
f1 (ppm)
OH
O
2

p-Coumaroyl meldrum's 1H

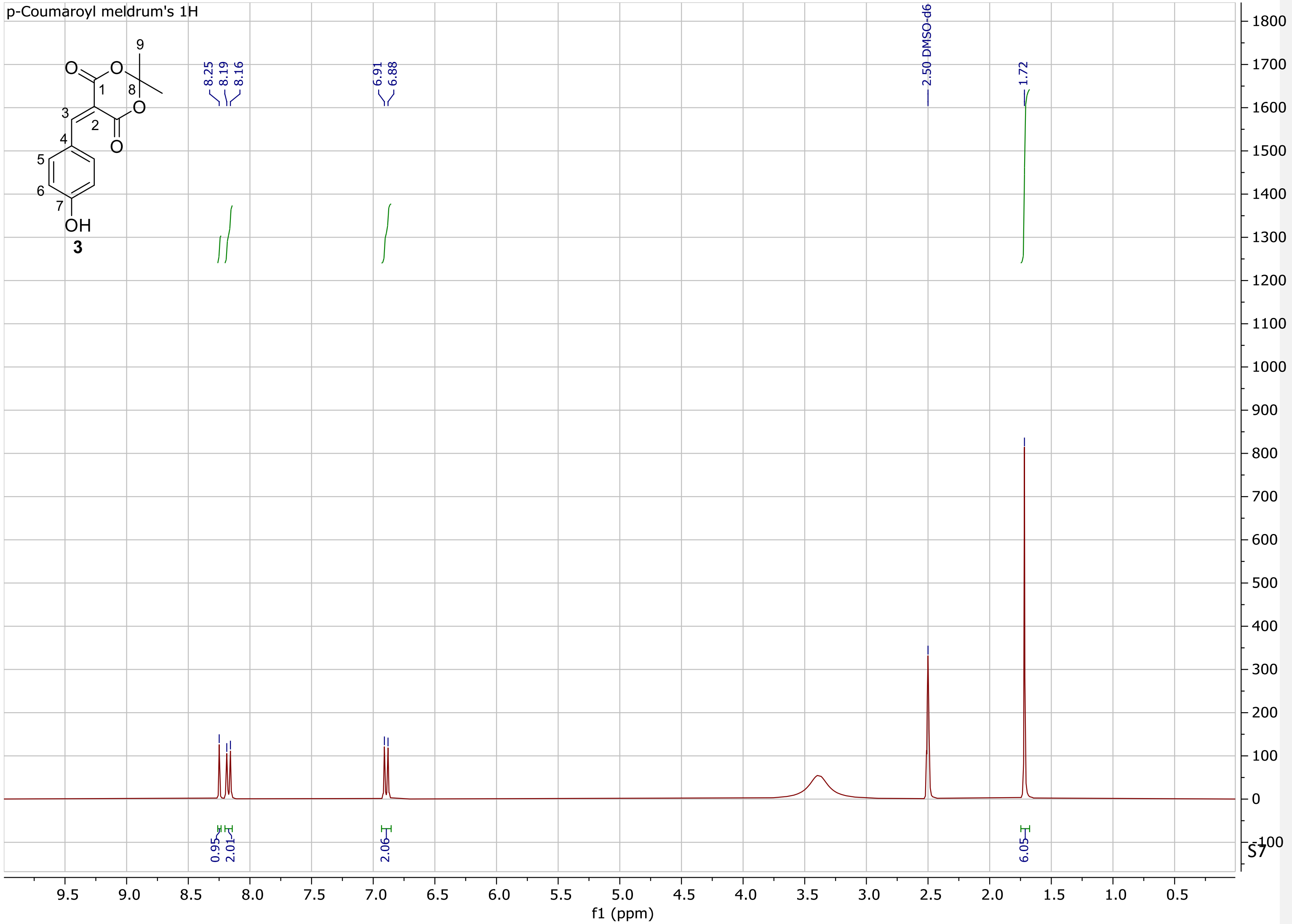

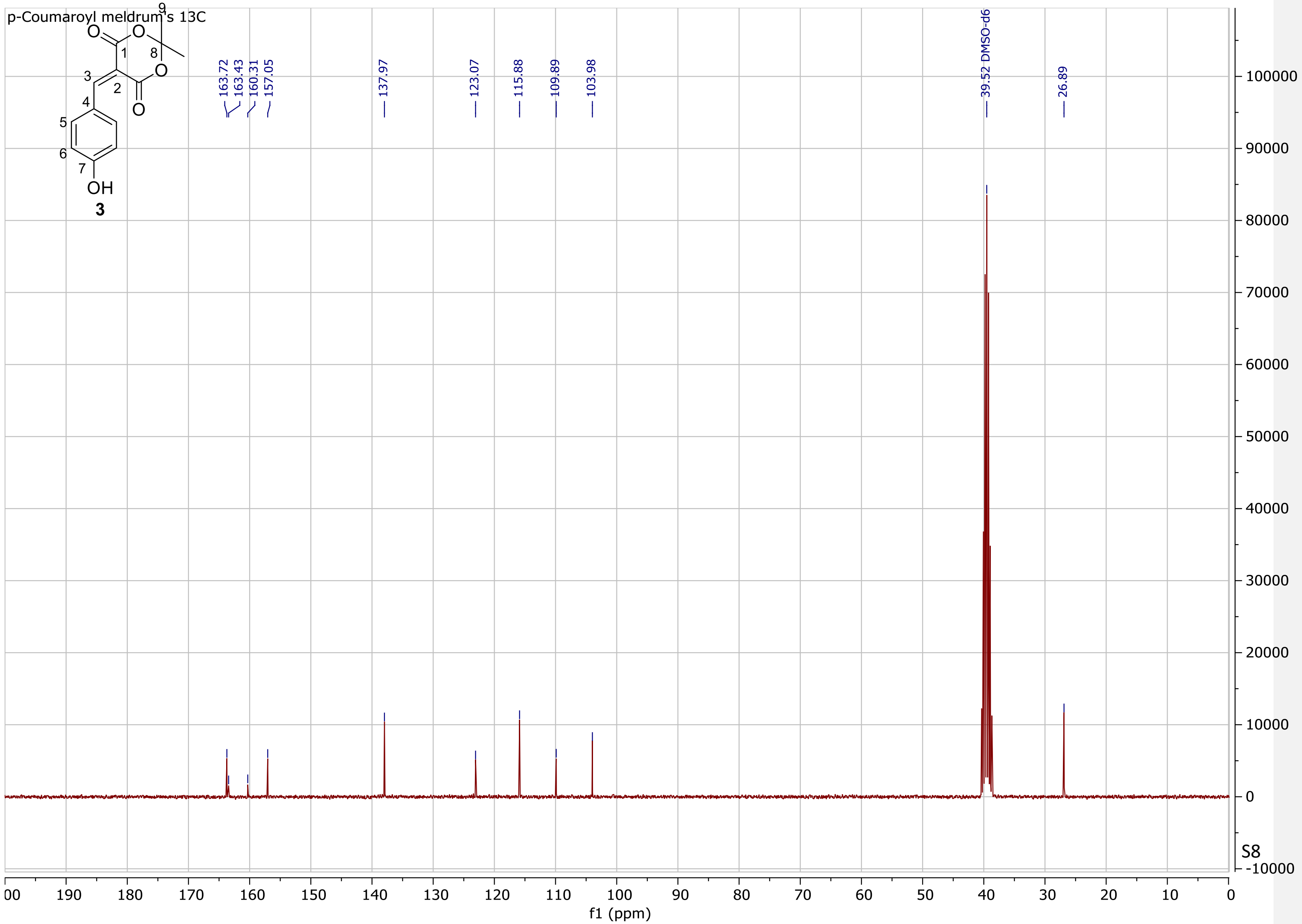
p-Coumaroyl meldrum's 13C
1
2
3
4
5
6
7
8
9
O
OH
3
163.72
163.43
160.31
157.05
137.97
123.07
115.88
109.89
103.98
39.52 DMSO-d6
26.89
f1 (ppm)

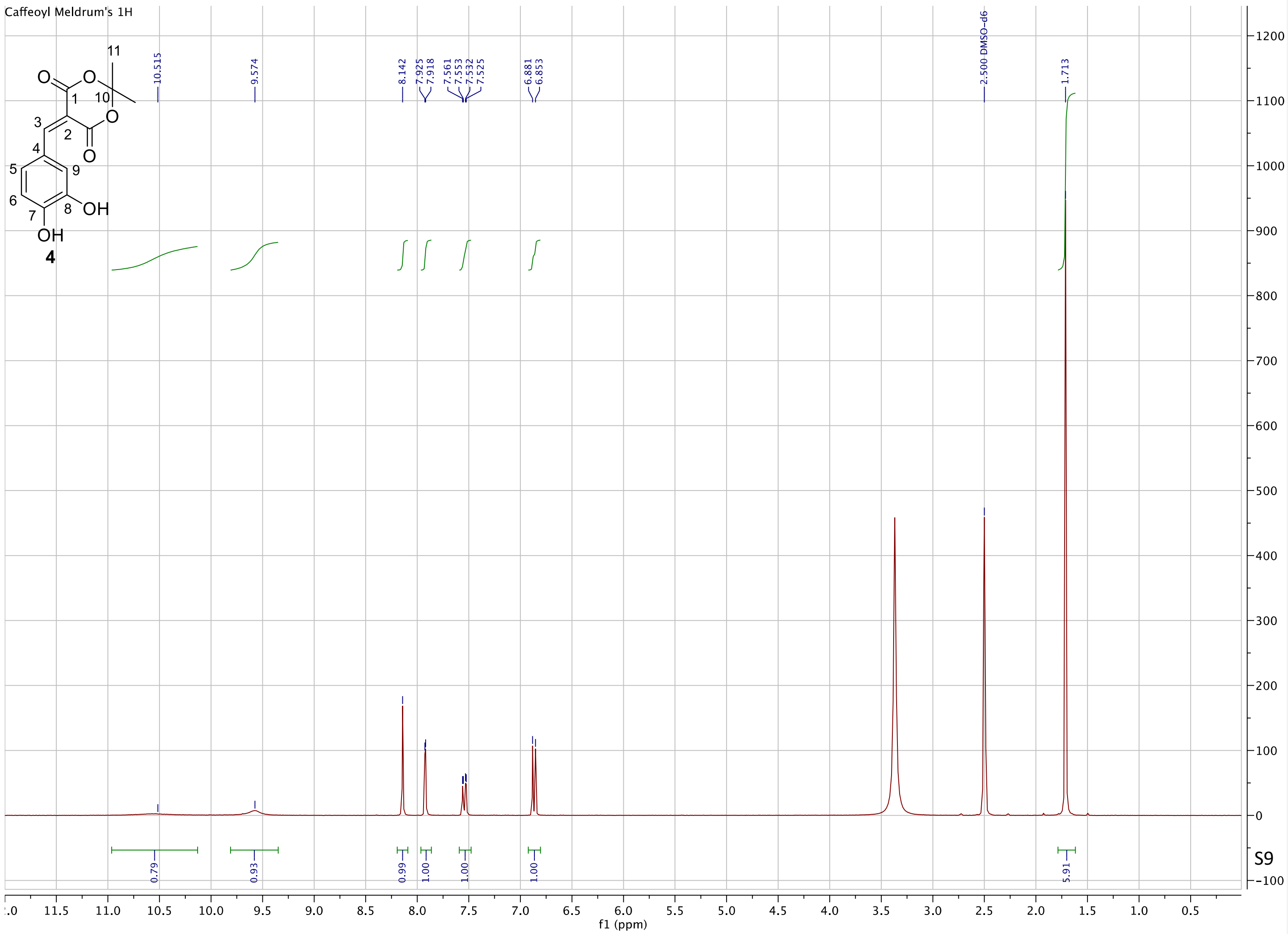
Caffeoyl Meldrum's 1H
4
10.515
9.574
8.142
7.925
7.918
7.561
7.553
7.532
7.525
6.881
6.853
2.500 DMSO-d6
1.713
0.79
0.93
0.99
1.00
1.00
1.00
5.91
f1 (ppm)

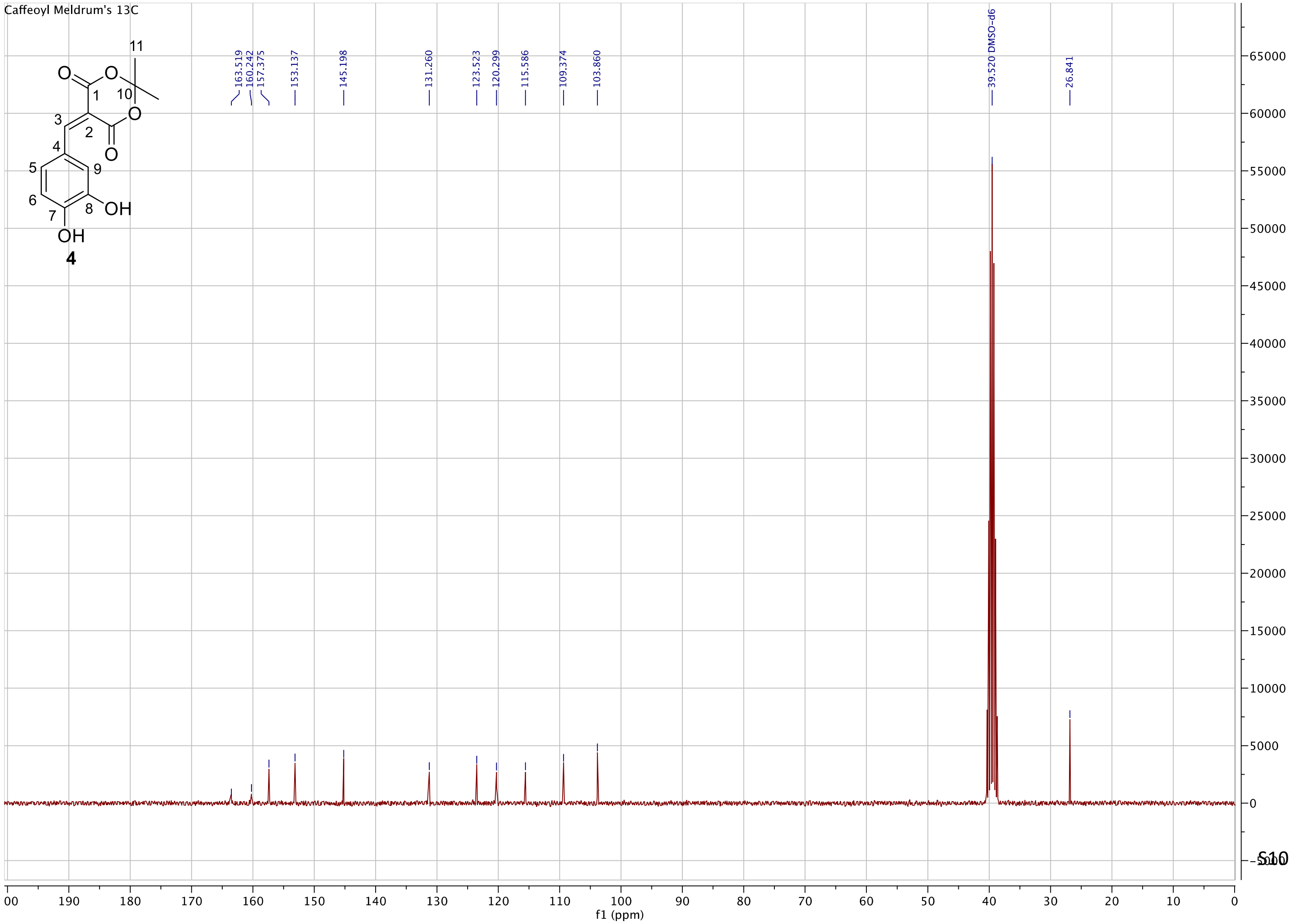
Caffeoyl Meldrum's 13C
4
163.519
160.242
157.375
153.137
145.198
131.260
123.523
120.299
115.586
109.374
103.860
39.520 DMSO-d6
26.841
f1 (ppm)

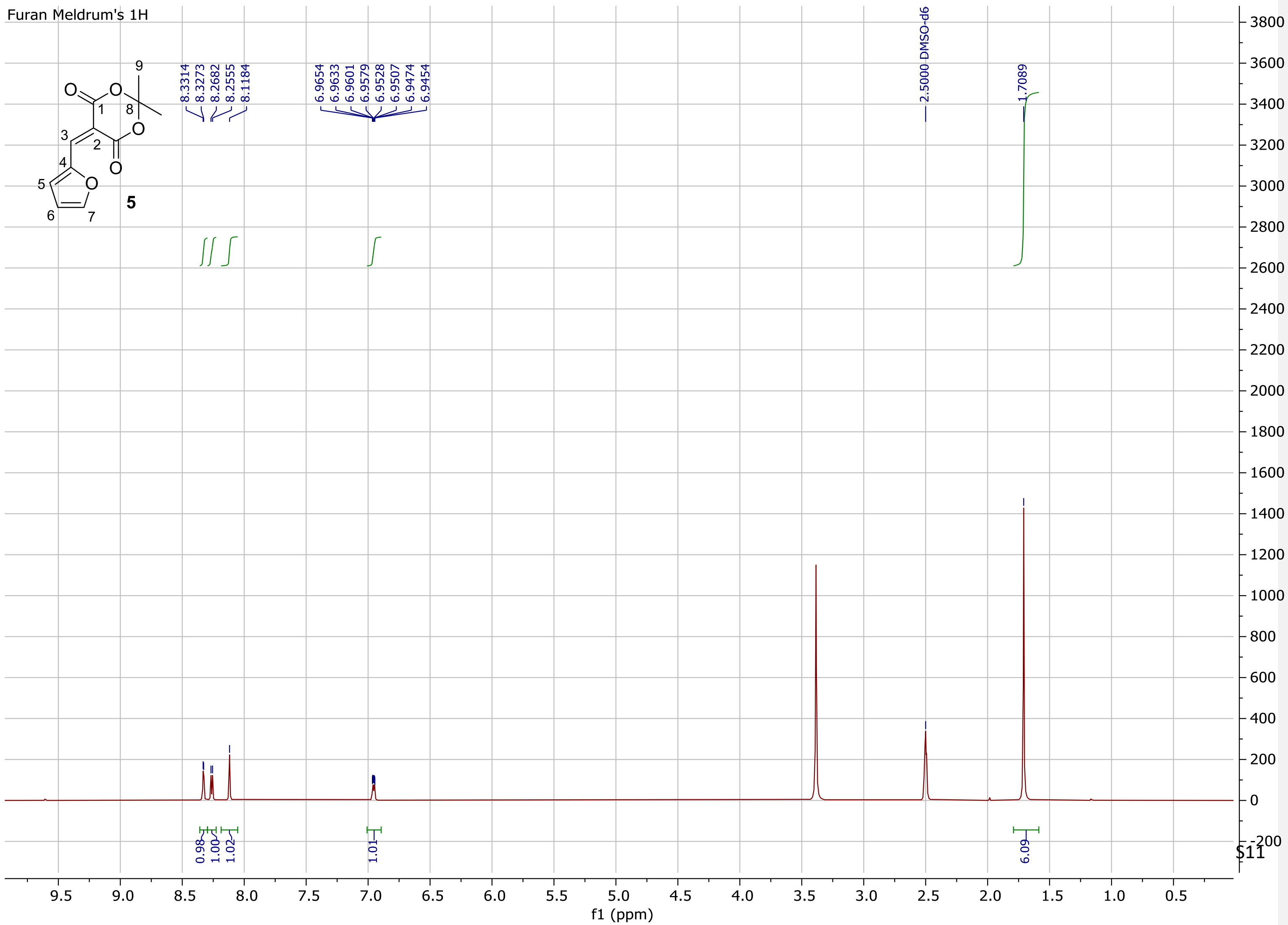
Furan Meldrum's 1H
9
8
1
2
3
4
5
6
7
5
8.3314
8.3273
8.2682
8.2555
8.1184
6.9654
6.9633
6.9601
6.9579
6.9528
6.9507
6.9474
6.9454
2.5000 DMSO-d6
1.7089
0.98
1.00
1.02
1.01
6.09
f1 (ppm)

Furan Meldrum's 13C

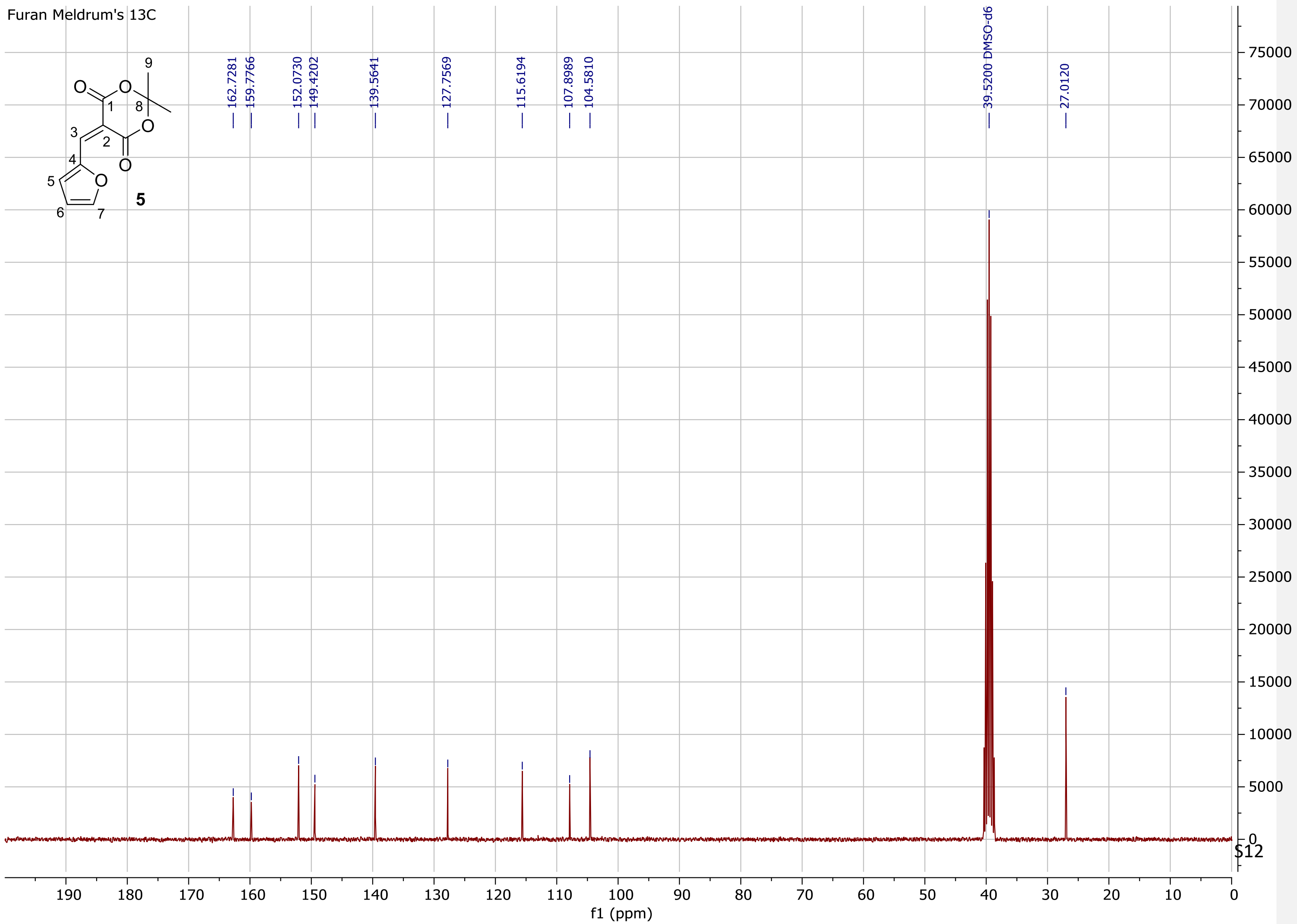

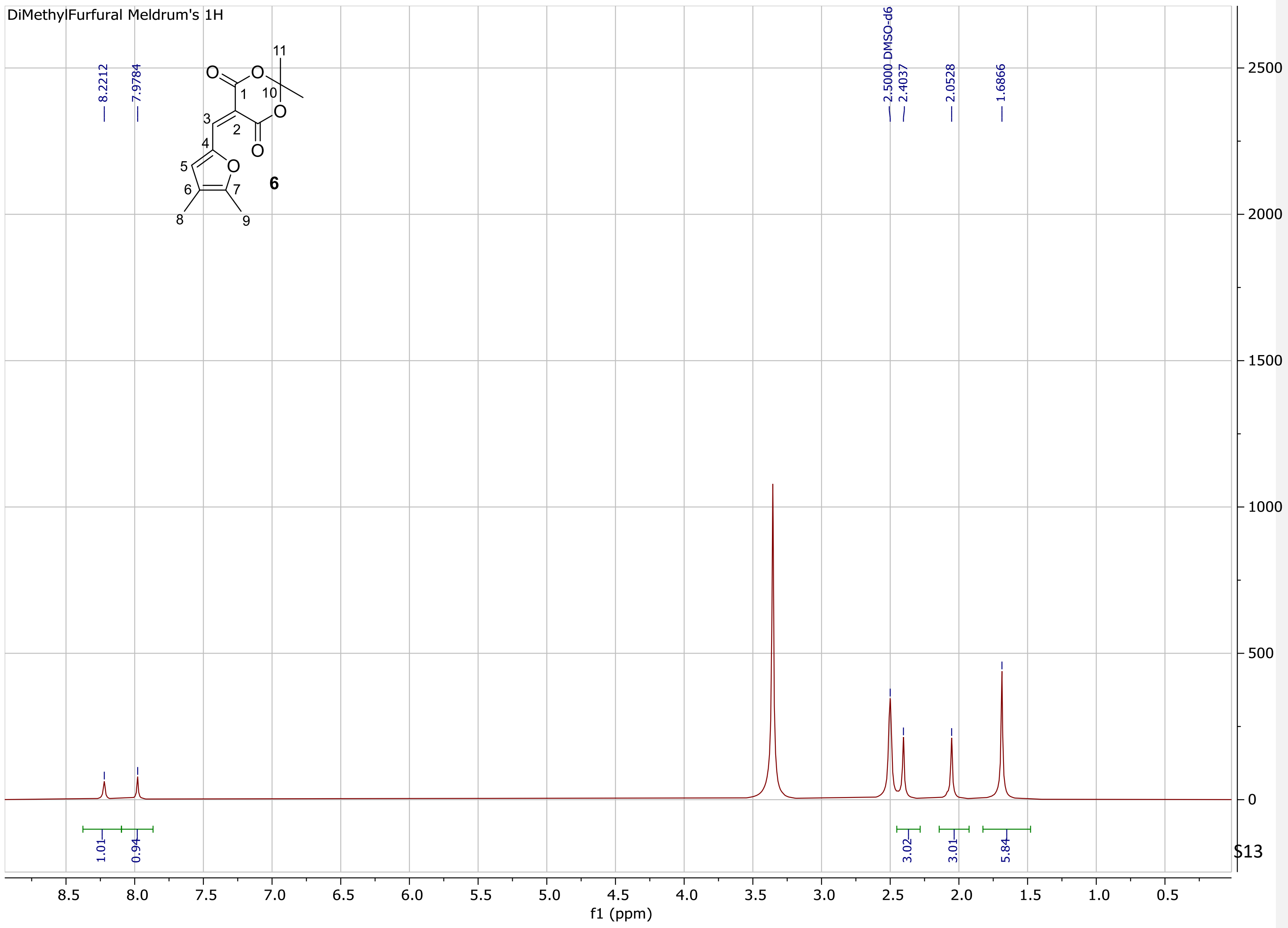
DiMethylFurfural Meldrum's 1H
6
8.2212
7.9784
2.5000 DMSO-d6
2.4037
2.0528
1.6866
1.01
0.94
3.02
3.01
5.84
f1 (ppm)

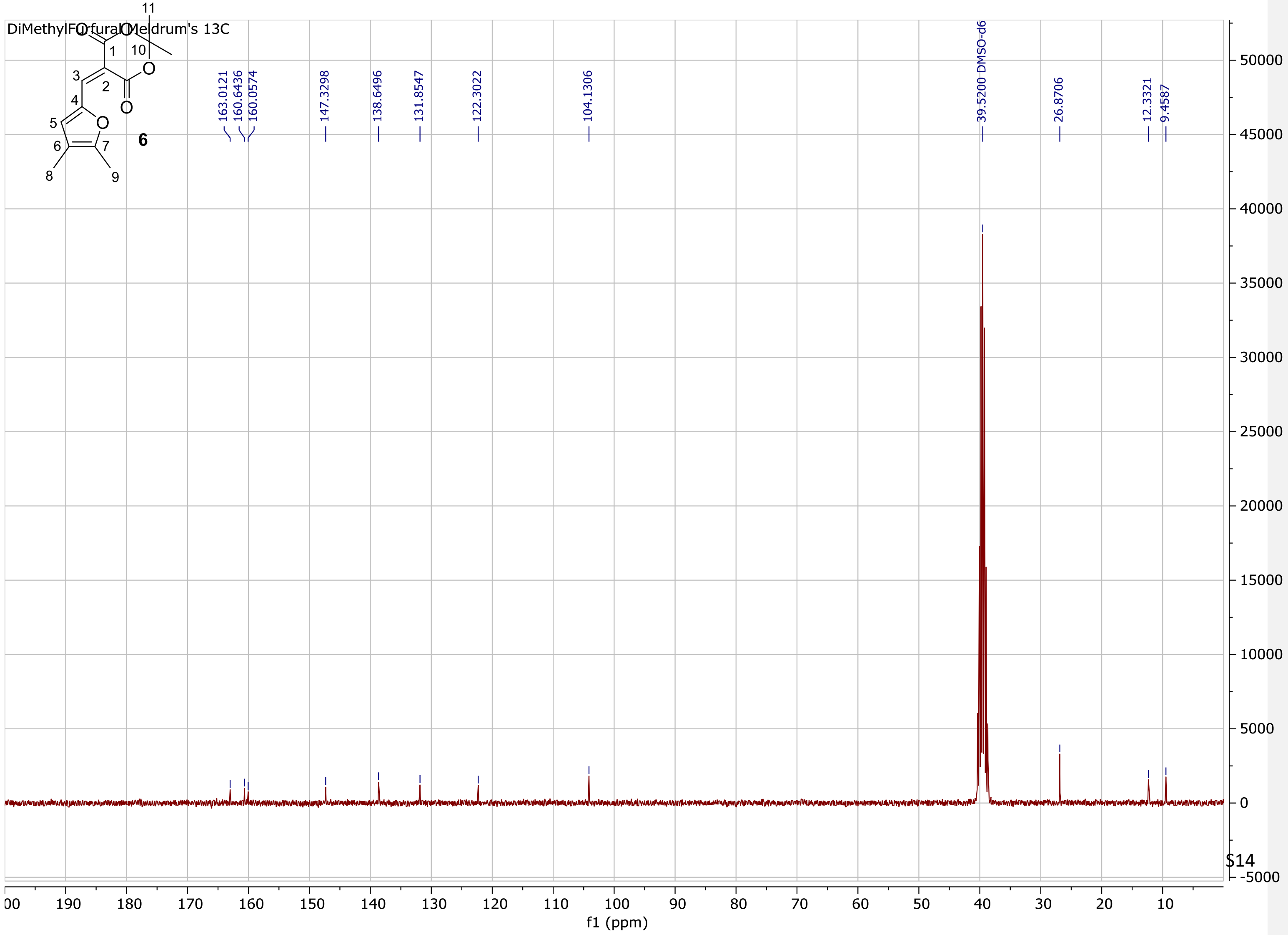
DiMethylFurfuralMeldrum's 13C
6
163.0121
160.6436
160.0574
147.3298
138.6496
131.8547
122.3022
104.1306
39.5200 DMSO-d6
26.8706
12.3321
9.4587
f1 (ppm)

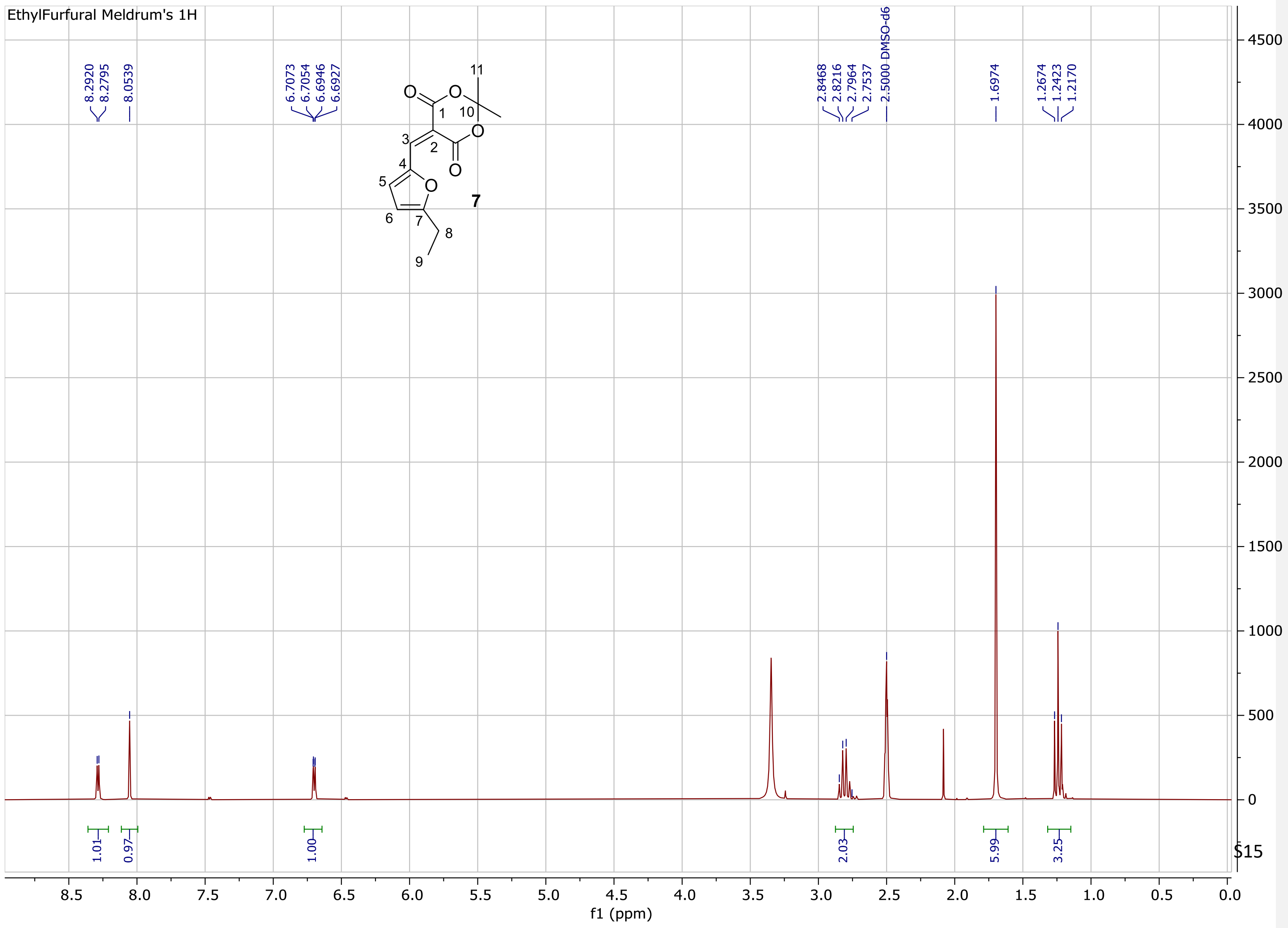
EthylFurfural Meldrum's 1H
8.2920
8.2795
8.0539
6.7073
6.7054
6.6946
6.6927
2.8468
2.8216
2.7964
2.7537
2.5000 DMSO-d6
1.6974
1.2674
1.2423
1.2170
1.01
0.97
1.00
2.03
5.99
3.25
7
f1 (ppm)

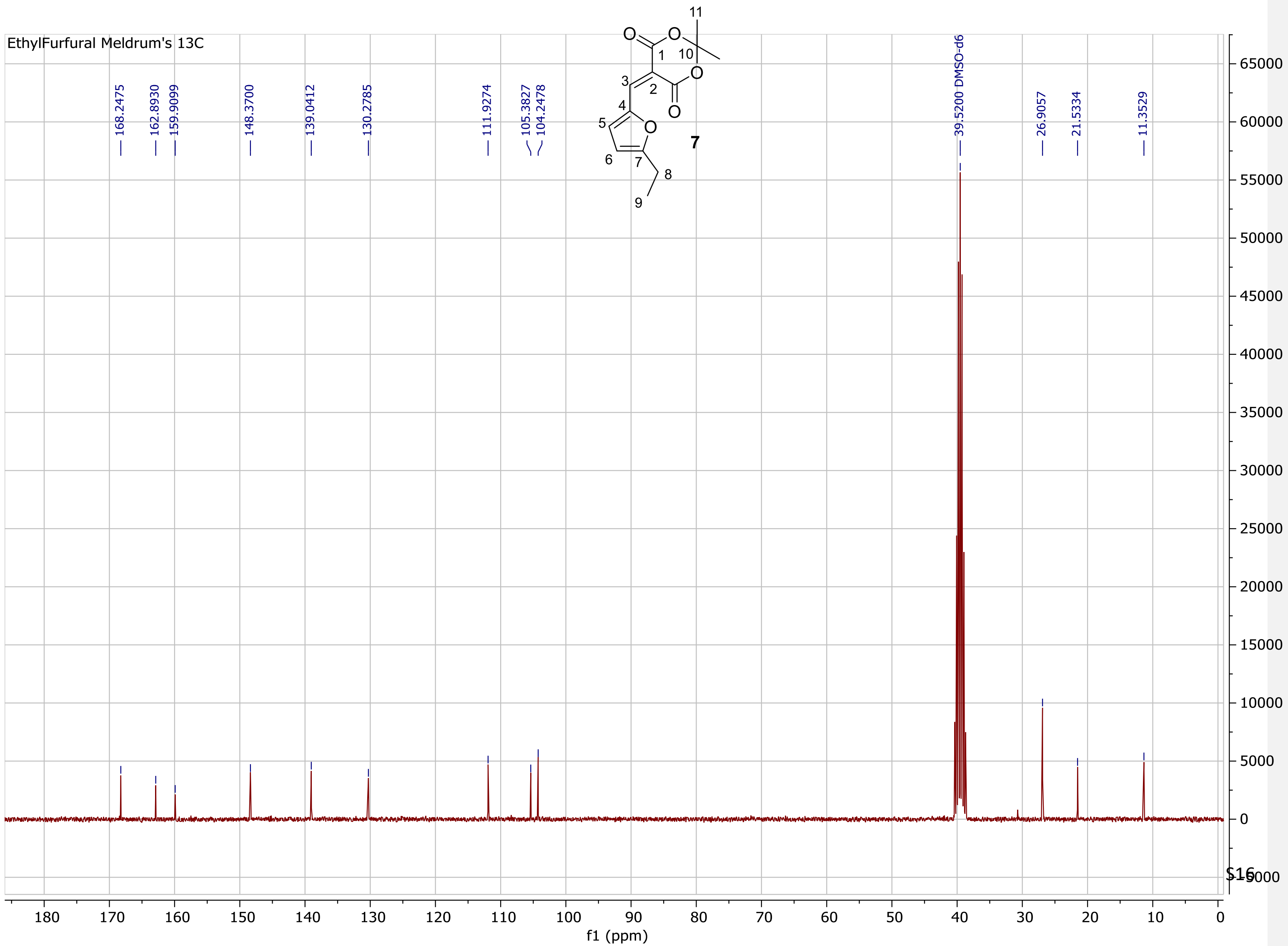
EthylFurfural Meldrum's 13C
168.2475
162.8930
159.9099
148.3700
139.0412
130.2785
111.9274
105.3827
104.2478
39.5200 DMSO-d6
26.9057
21.5334
11.3529
7
f1 (ppm)

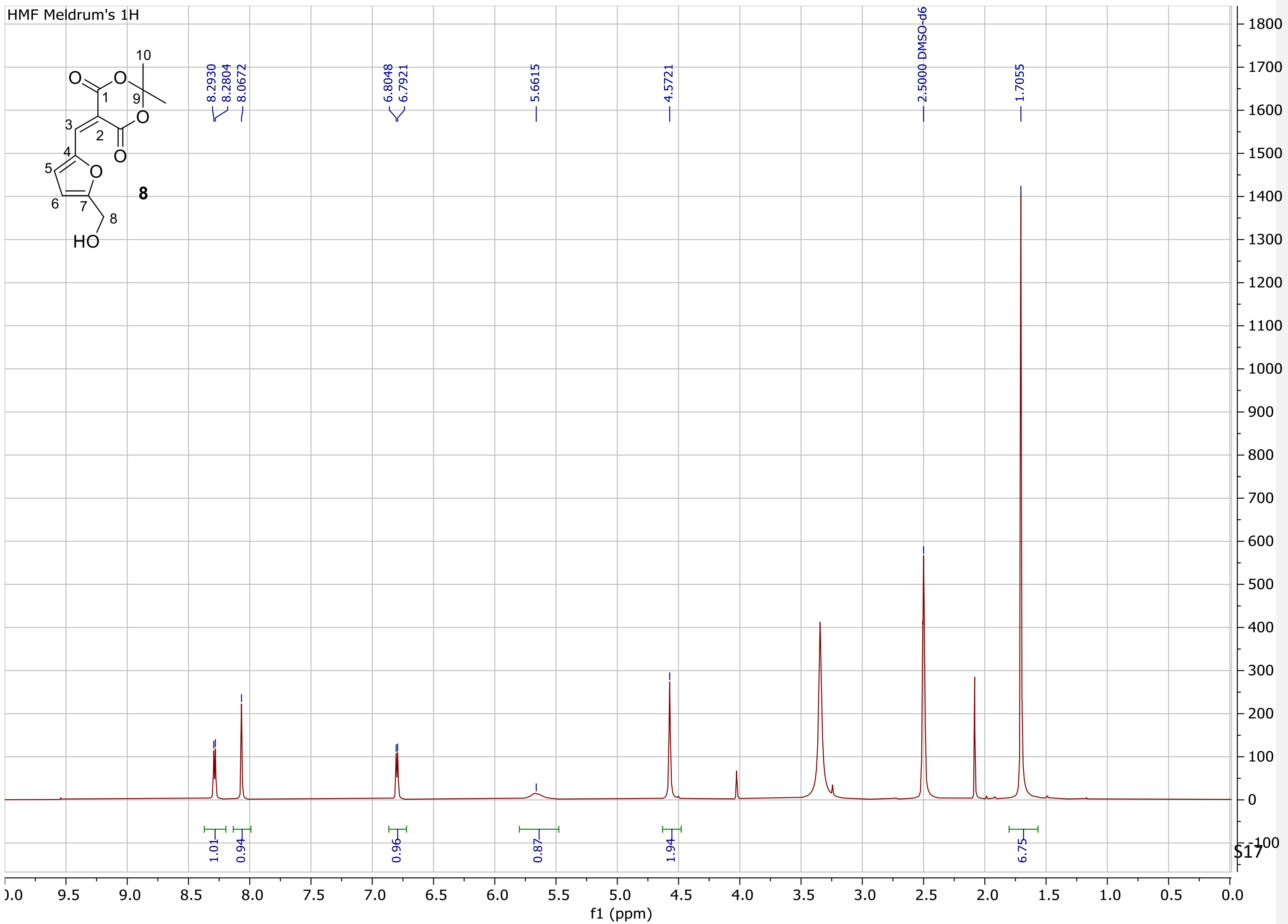
HMF Meldrum's 1H
8
8.2930
8.2804
8.0672
6.8048
6.7921
5.6615
4.5721
2.5000 DMSO-d6
1.7055
1.01
0.94
0.96
0.87
1.94
6.75
f1 (ppm)

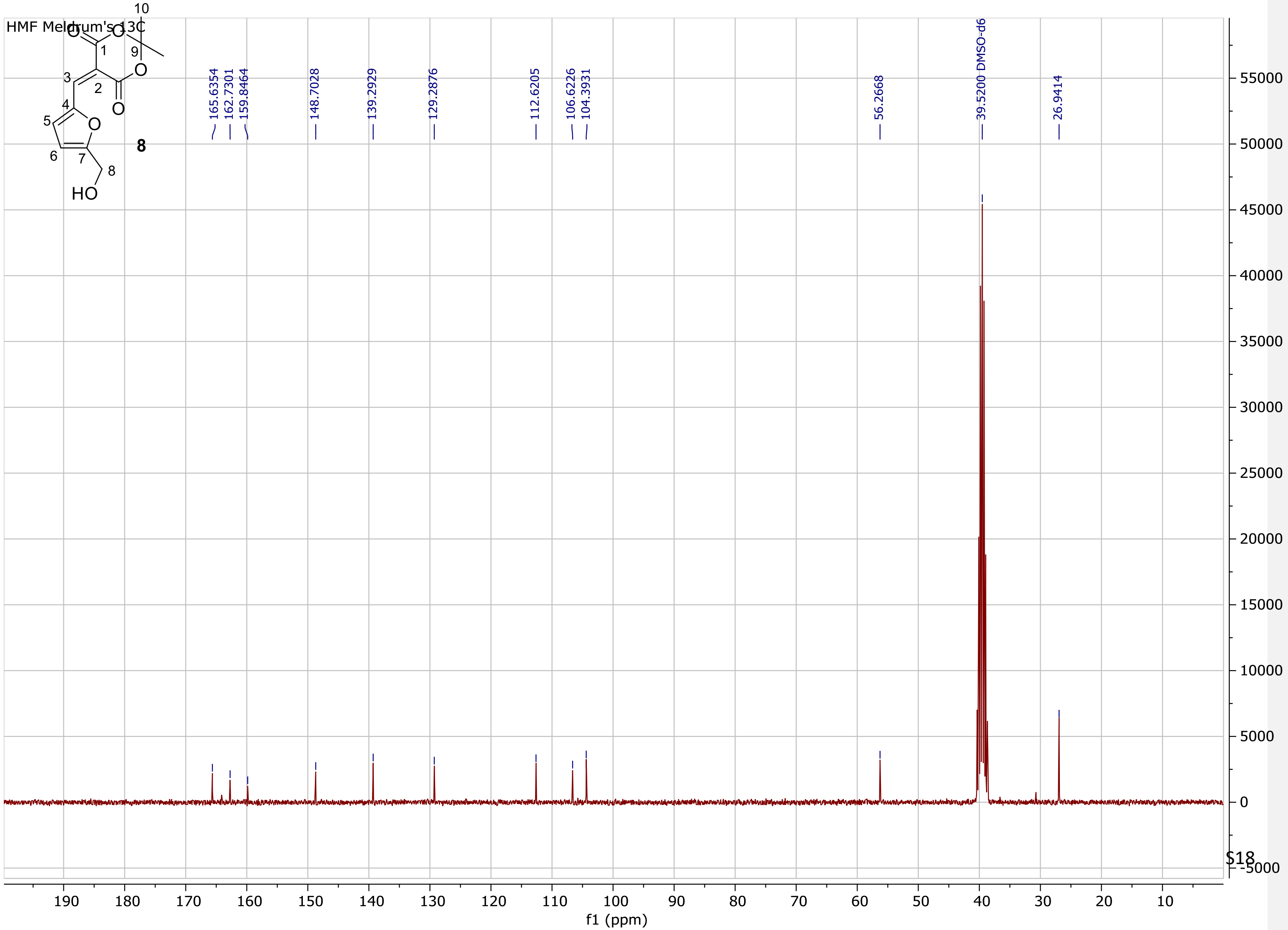

HMF Meldrum's 13C
10
9
1
2
3
4
5
6
7
8
O
HO
8
165.6354
162.7301
159.8464
148.7028
139.2929
129.2876
112.6205
106.6226
104.3931
56.2668
39.5200 DMSO-d6
26.9414
55000
50000
45000
40000
35000
30000
25000
20000
15000
10000
5000
0
-5000
190
180
170
160
150
140
130
120
110
100
90
80
70
60
50
40
30
20
10
f1 (ppm)

Benzofuran Meldrum's 1H

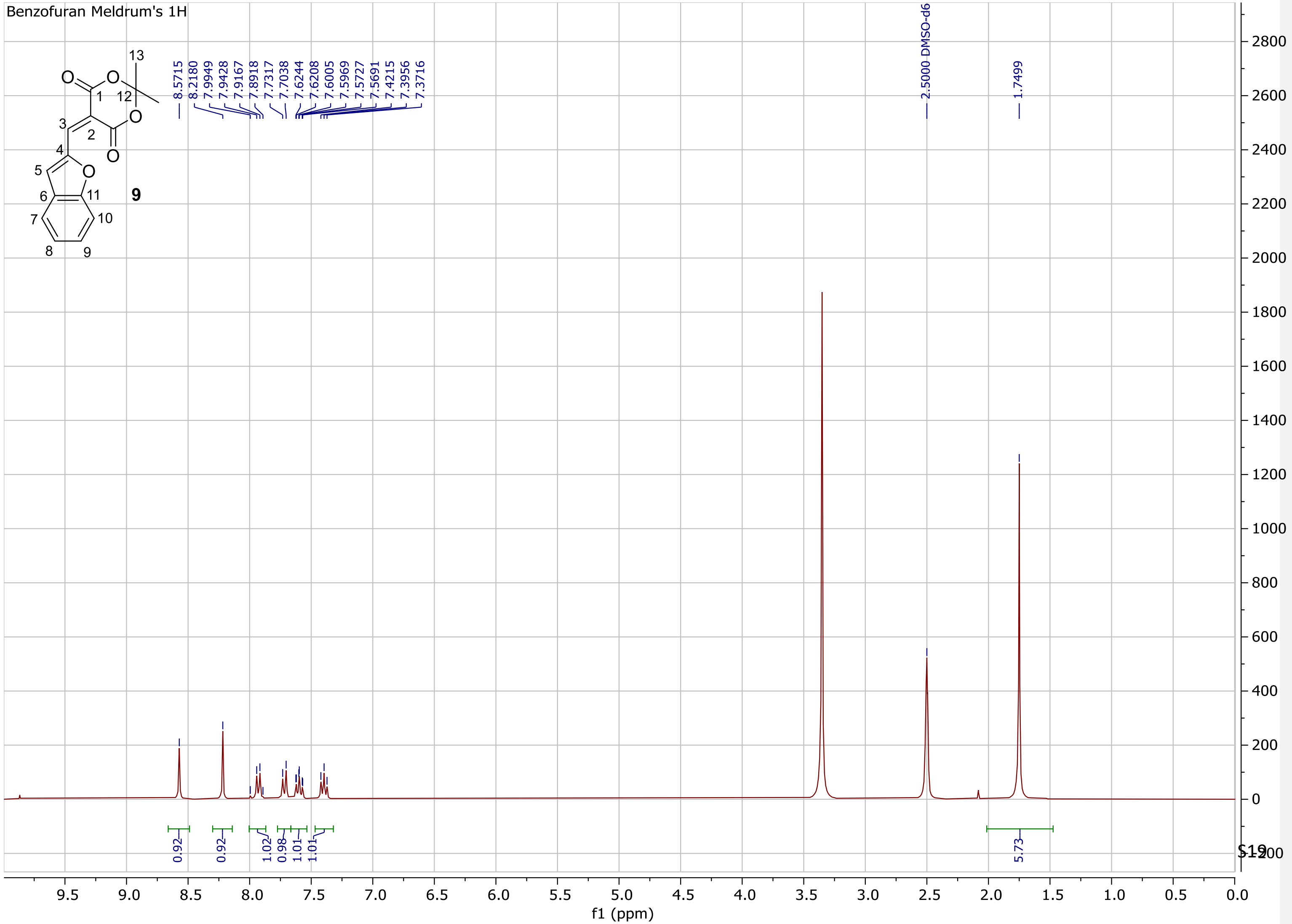

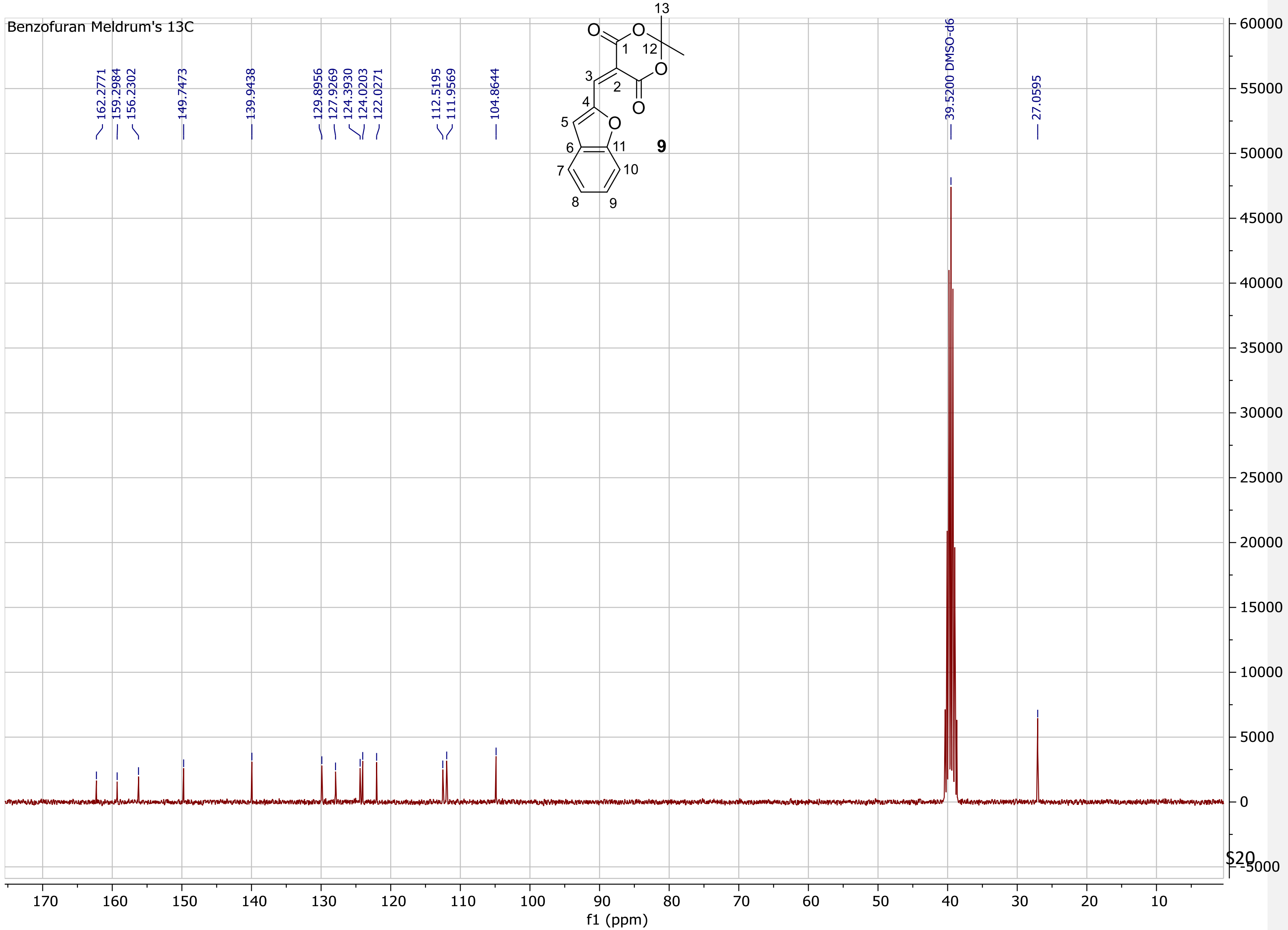

Benzofuran Meldrum's 13C
162.2771
159.2984
156.2302
149.7473
139.9438
129.8956
127.9269
124.3930
124.0203
122.0271
112.5195
111.9569
104.8644
39.5200 DMSO-d6
27.0595
9
f1 (ppm)

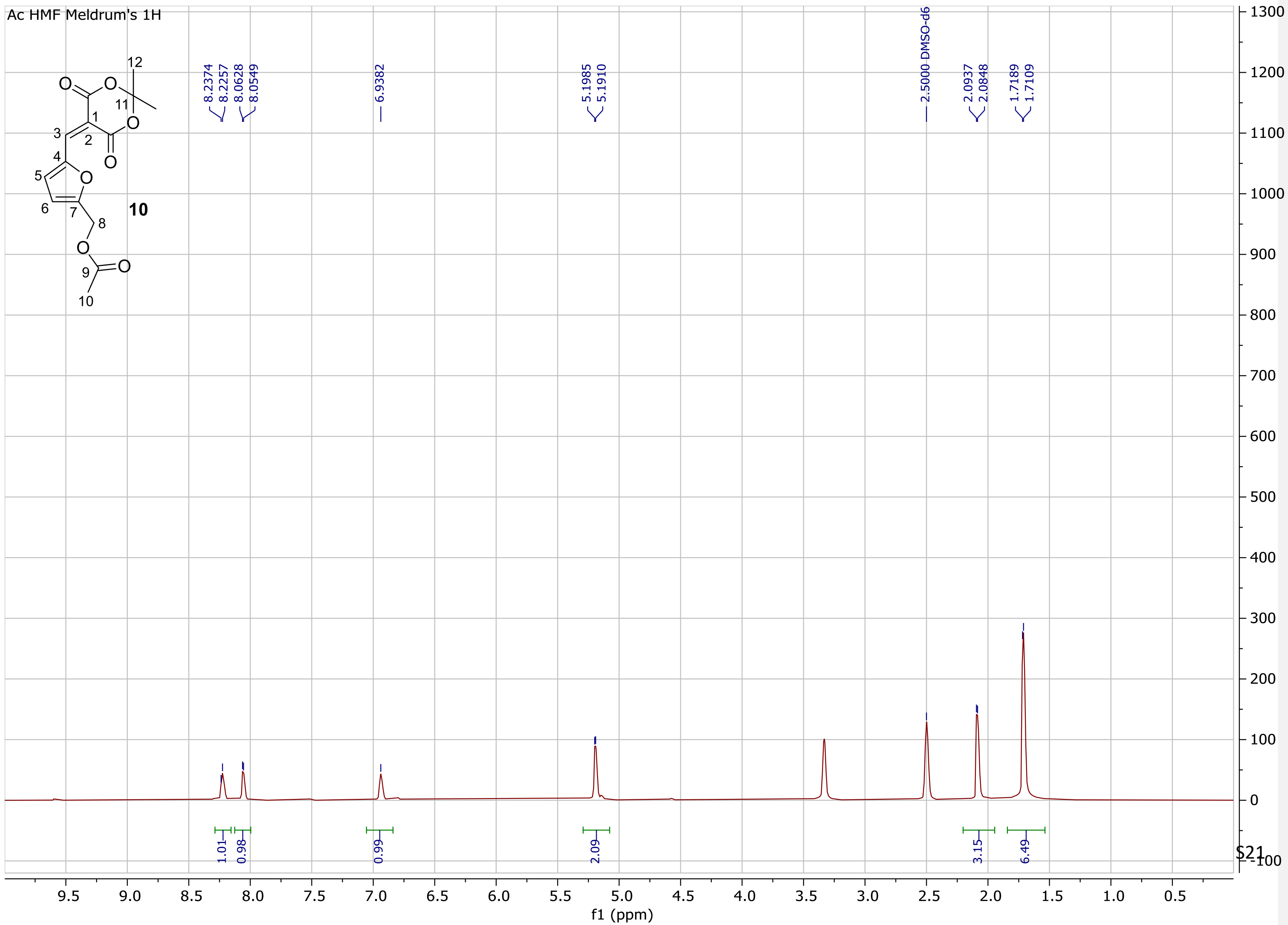
Ac HMF Meldrum's 1H
10
8.2374
8.2257
8.0628
8.0549
6.9382
5.1985
5.1910
2.5000 DMSO-d6
2.0937
2.0848
1.7189
1.7109
1.01
0.98
0.99
2.09
3.15
6.49
f1 (ppm)

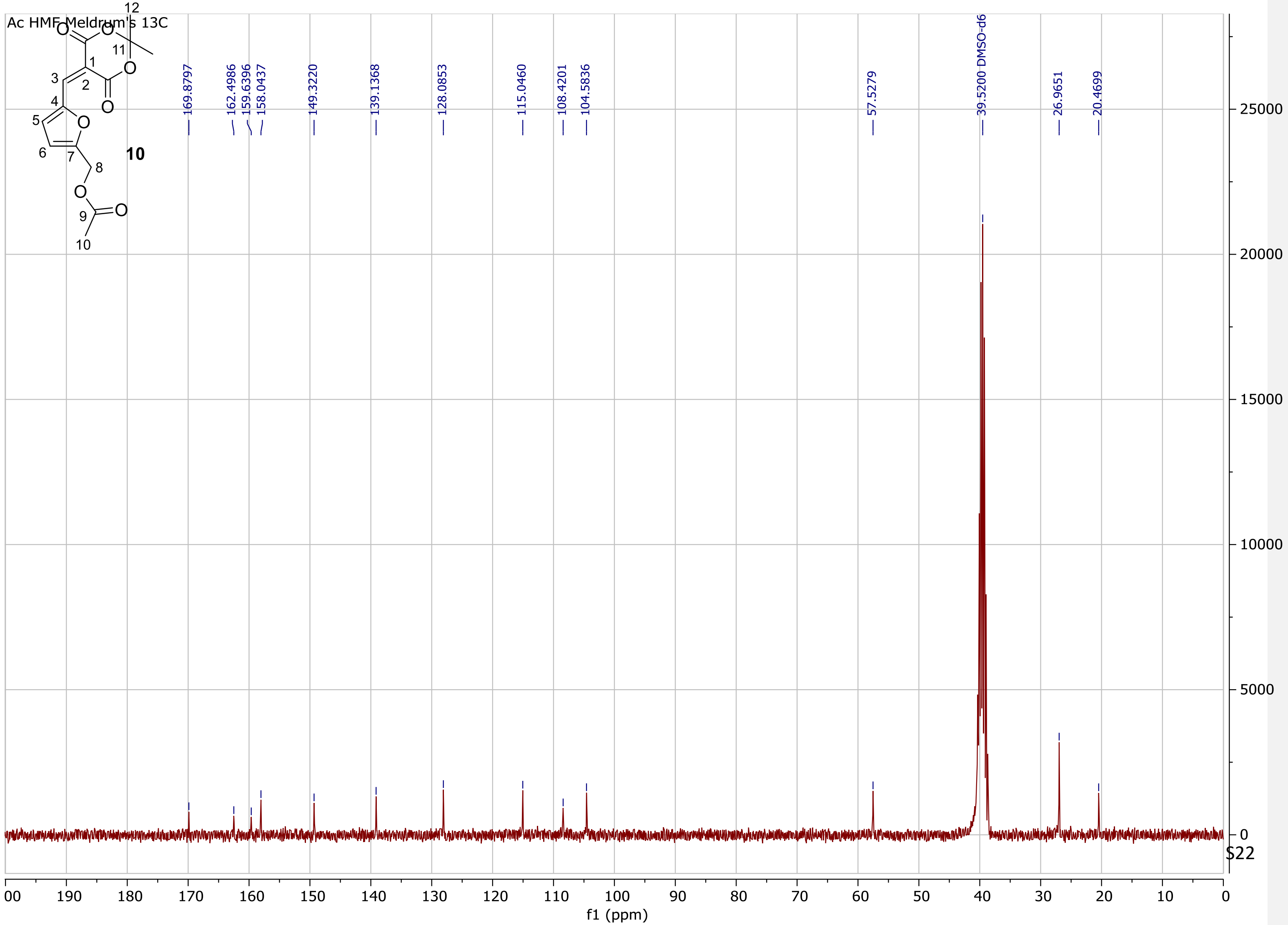

Ac HMF Meldrum's 13C
10
169.8797
162.4986
159.6396
158.0437
149.3220
139.1368
128.0853
115.0460
108.4201
104.5836
57.5279
39.5200 DMSO-d6
26.9651
20.4699
f1 (ppm)

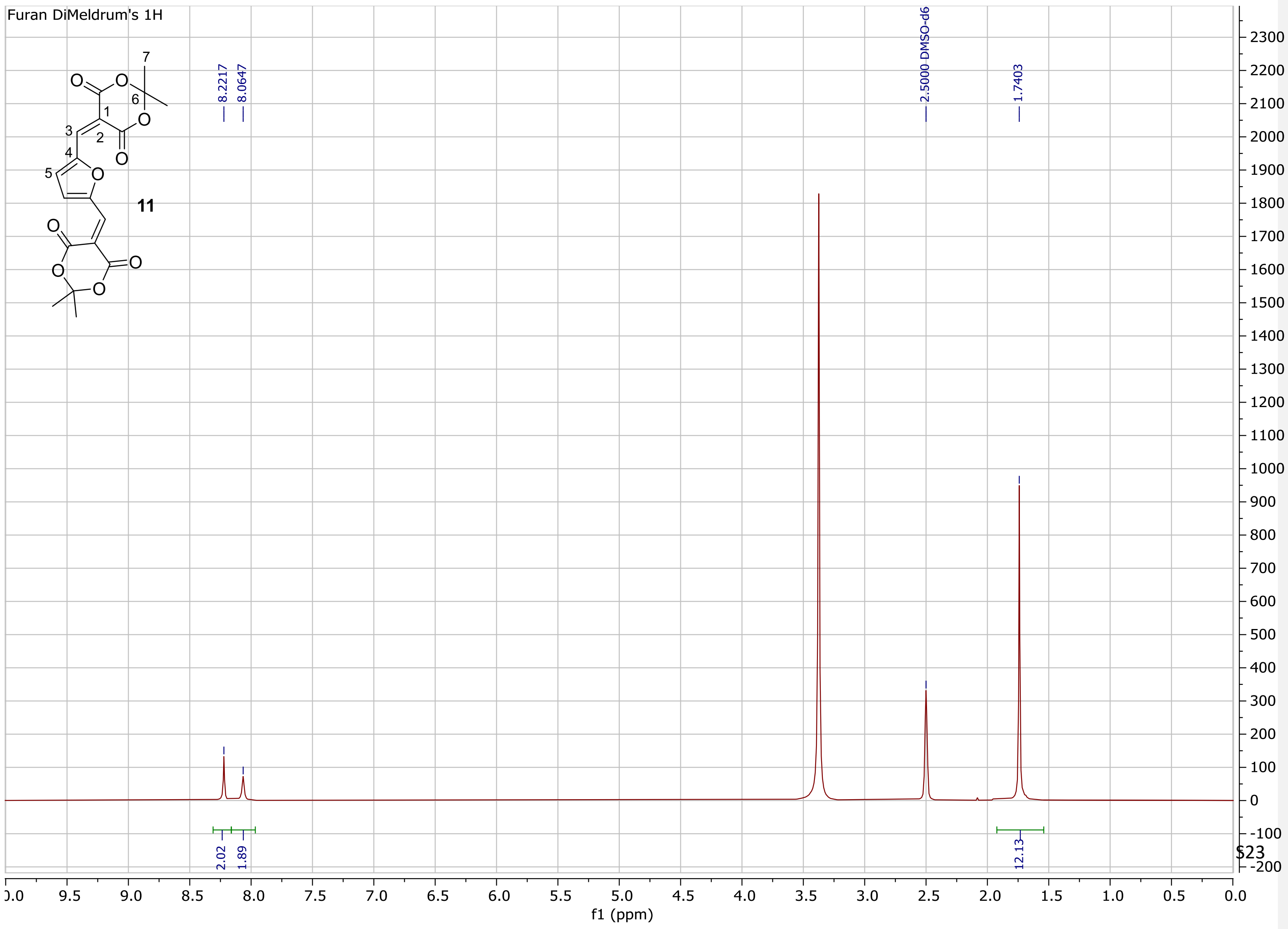

Furan DiMeldrum's 1H
11
8.2217
8.0647
2.5000 DMSO-d6
1.7403
2.02
1.89
12.13
f1 (ppm)

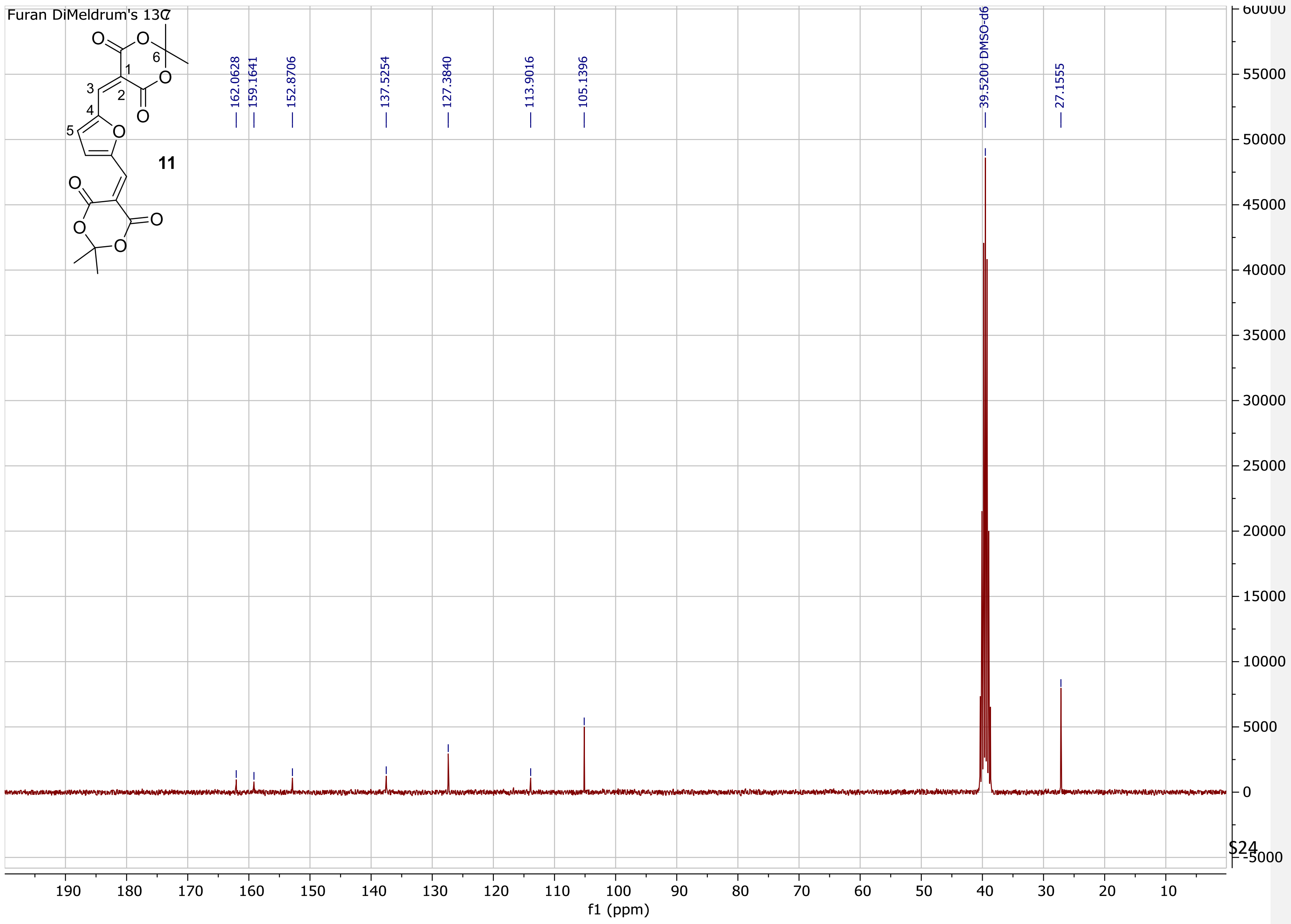
Furan DiMeldrum's 13C
11
162.0628
159.1641
152.8706
137.5254
127.3840
113.9016
105.1396
39.5200 DMSO-d6
27.1555
f1 (ppm)

2. UV Spectra

2.1. Phenolic series

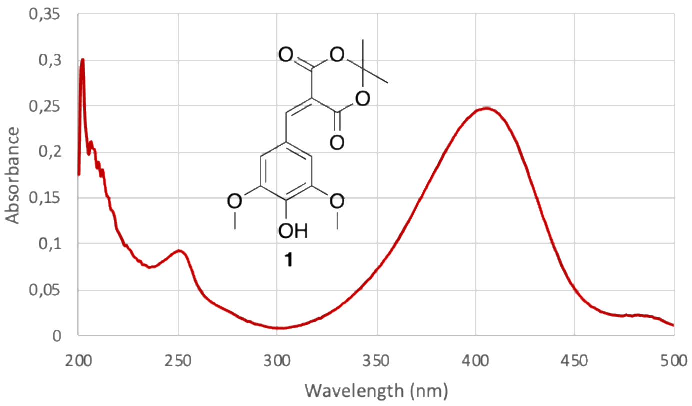

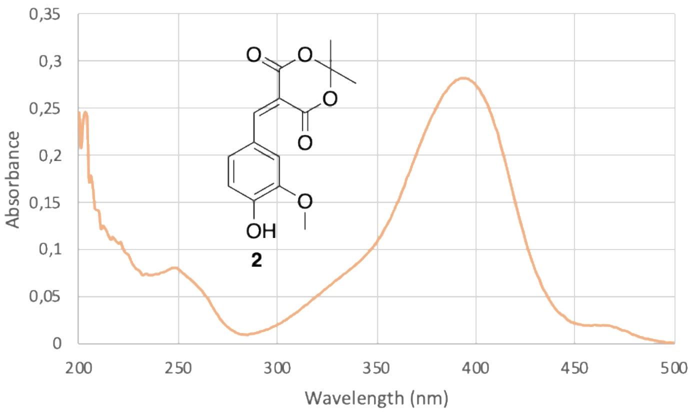

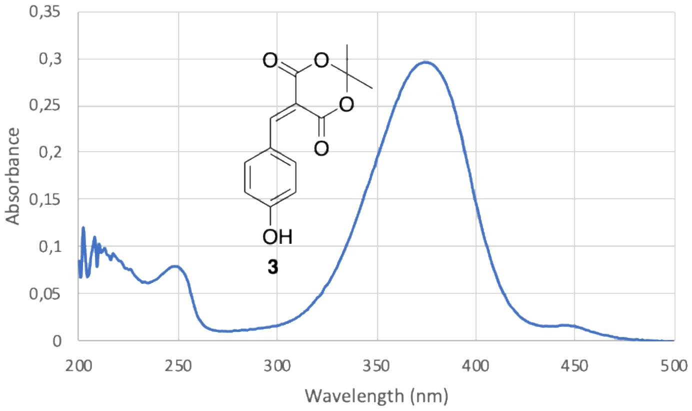

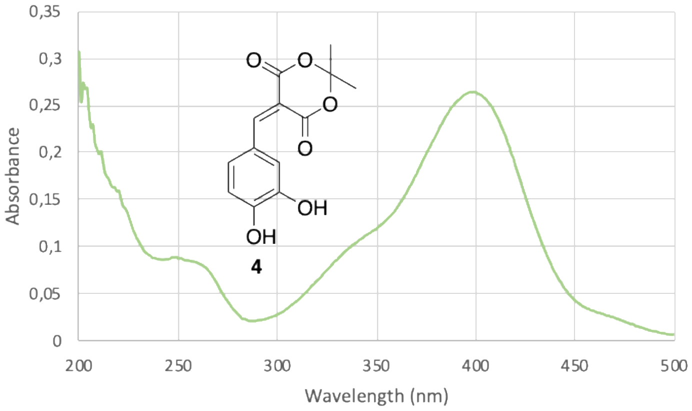

2.2. Furanic series

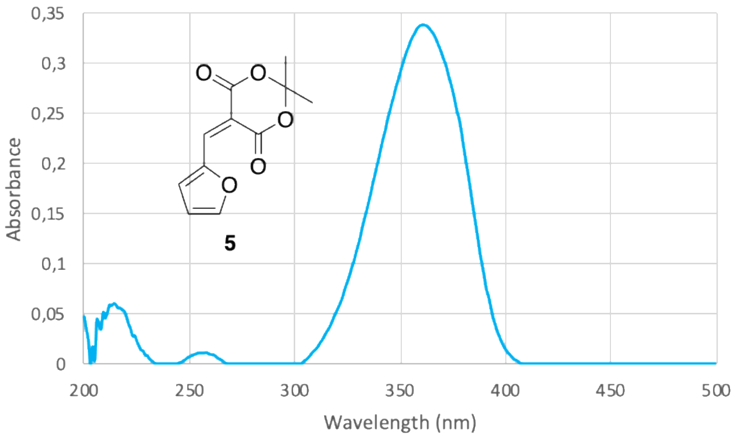

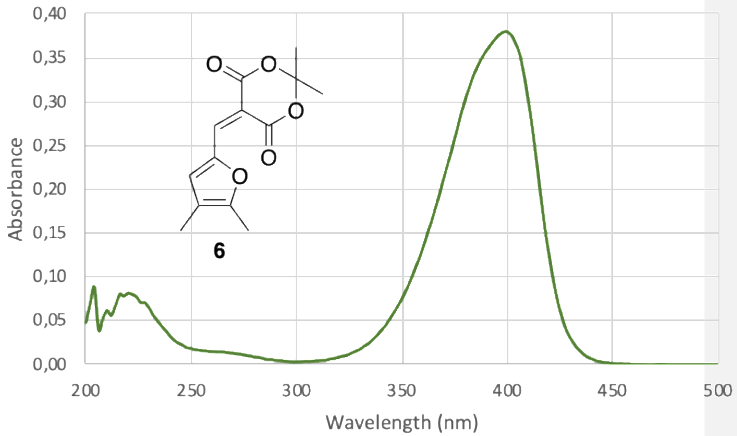

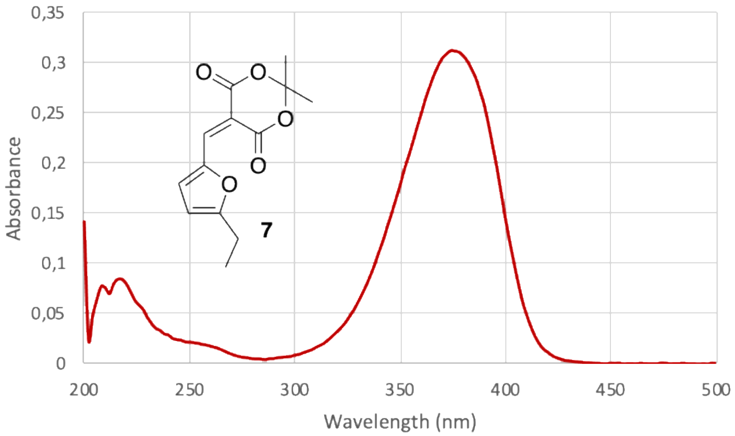

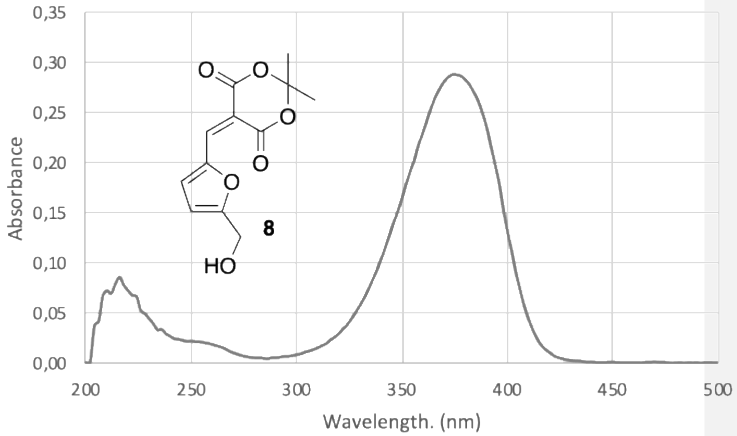

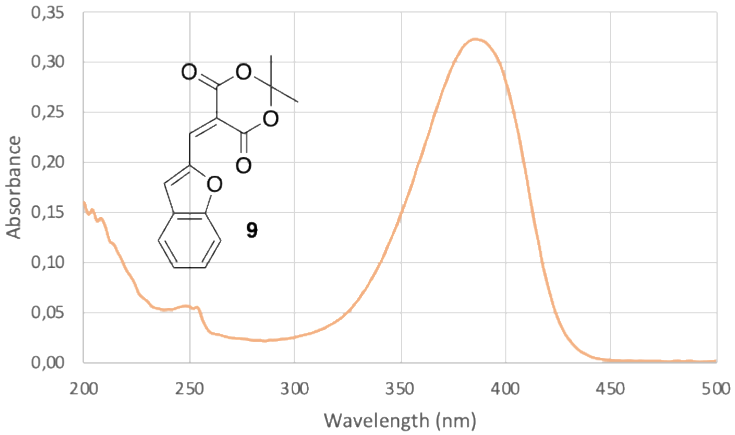

0,35
0,30
0,25
0,20
0,15
0,10
0,05
0,00
Absorbance
200
250
300
350
400
450
500
Wavelength (nm)
9

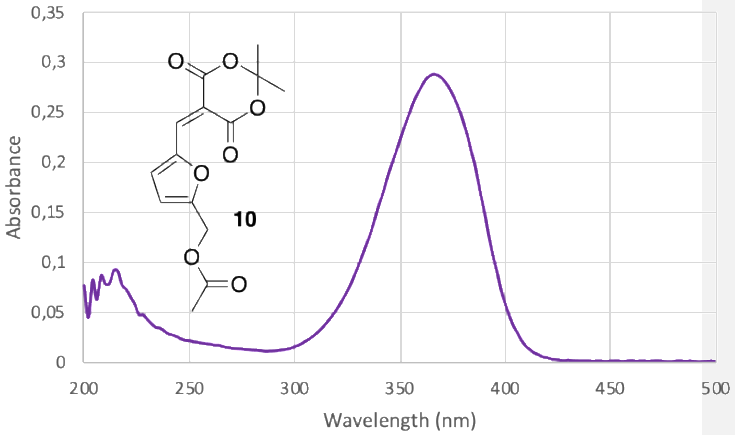

0,35
0,3
0,25
0,2
0,15
0,1
0,05
0
Absorbance
200
250
300
350
400
450
500
Wavelength (nm)
10

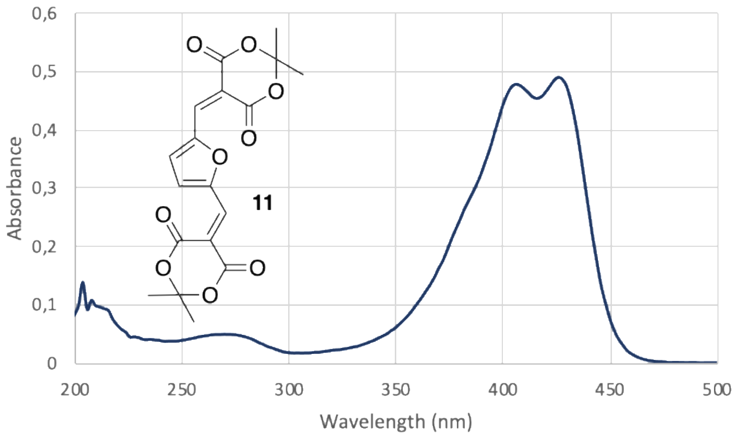

0,6
0,5
0,4
0,3
0,2
0,1
0
Absorbance
200
250
300
350
400
450
500
Wavelength (nm)
11

2.3. Pyrrolic series

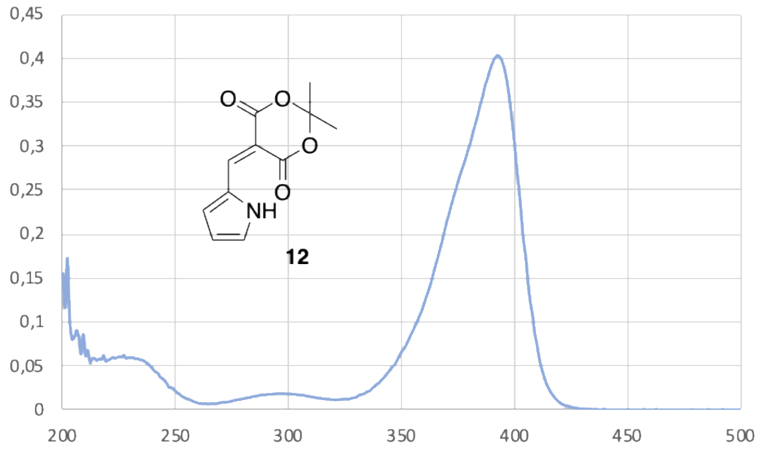

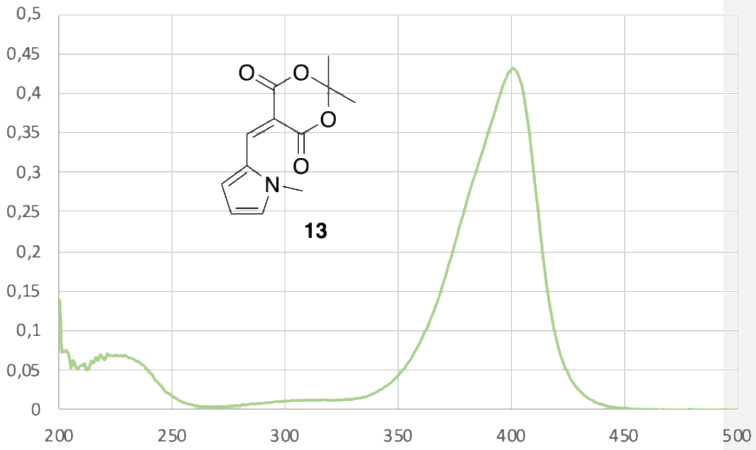

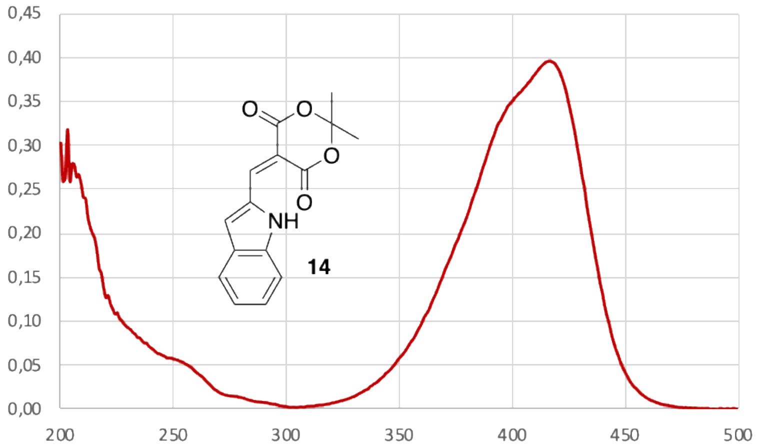

2.4. Mixtures

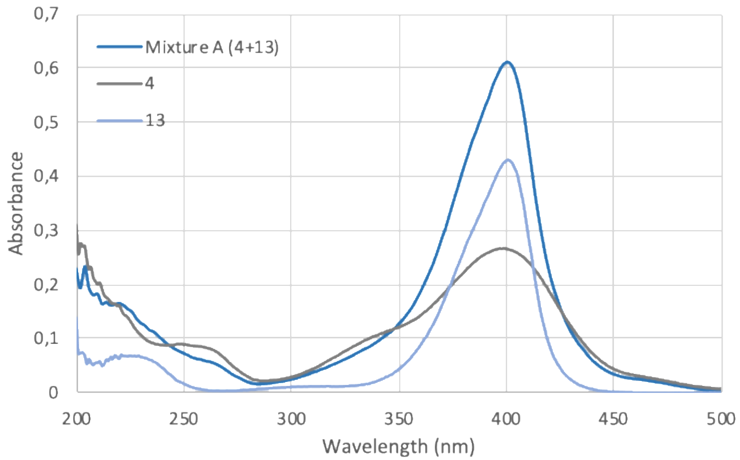
Mixture A (4+13)
4
13
Absorbance
Wavelength (nm)
0,7
0,6
0,5
0,4
0,3
0,2
0,1
0
200
250
300
350
400
450
500

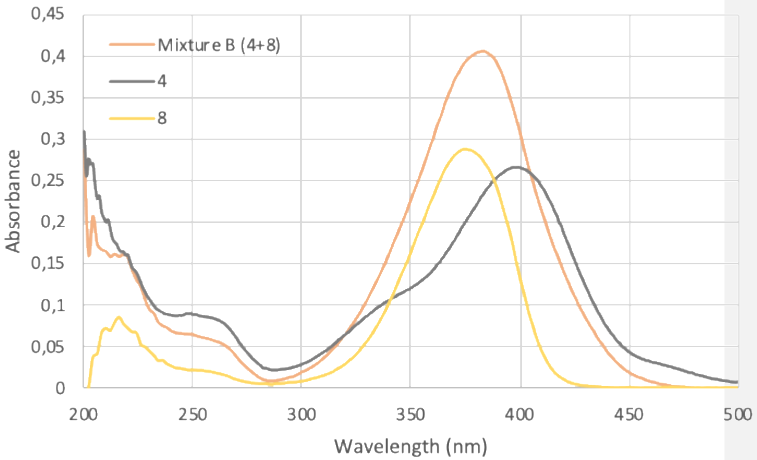
Mixture B (4+8)
4
8
Absorbance
Wavelength (nm)
0,45
0,4
0,35
0,3
0,25
0,2
0,15
0,1
0,05
0
200
250
300
350
400
450
500

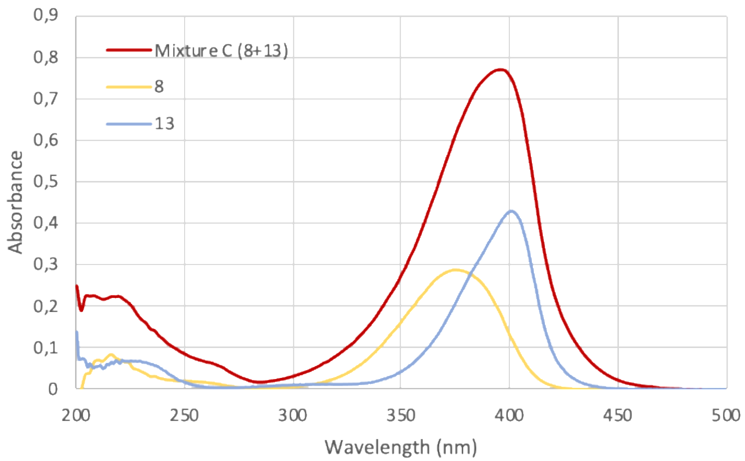
Mixture C (8+13)
8
13
Absorbance
Wavelength (nm)
0,9
0,8
0,7
0,6
0,5
0,4
0,3
0,2
0,1
0
200
250
300
350
400
450
500

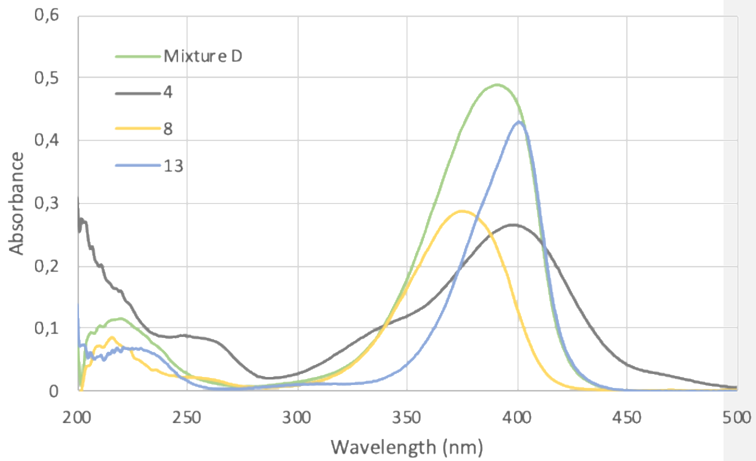
Mixture D
4
8
13
Absorbance
Wavelength (nm)
0,6
0,5
0,4
0,3
0,2
0,1
0
200
250
300
350
400
450
500

2.5. Reference

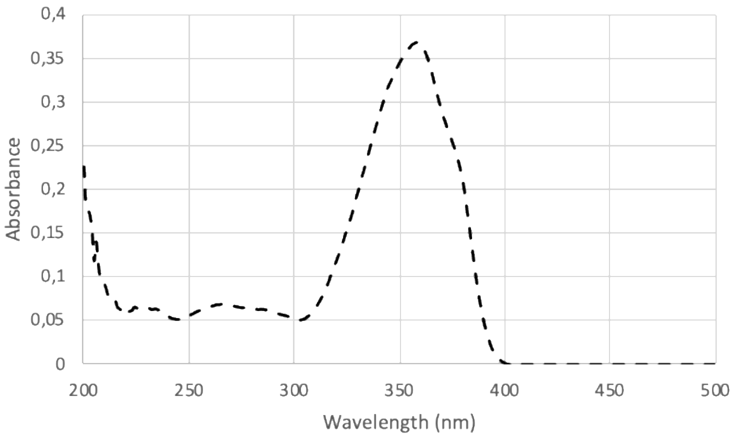

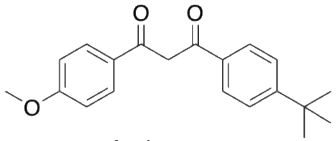

Avobenzone

3. Loss of absorbance (LoA)

3.1. Phenolic series

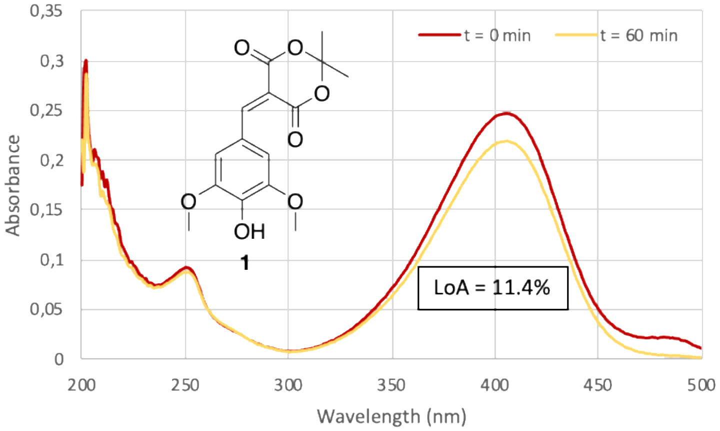

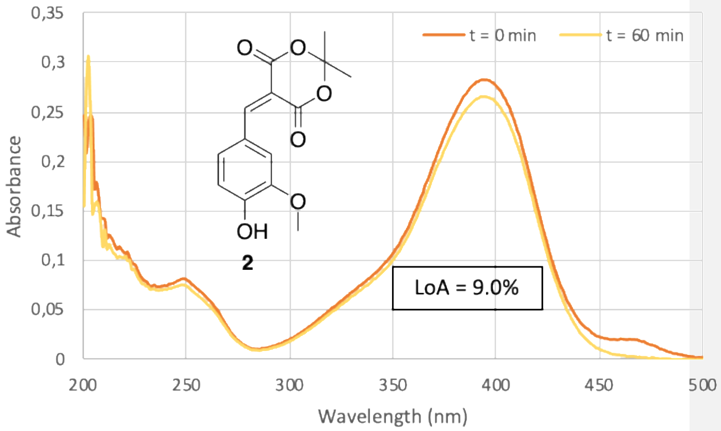

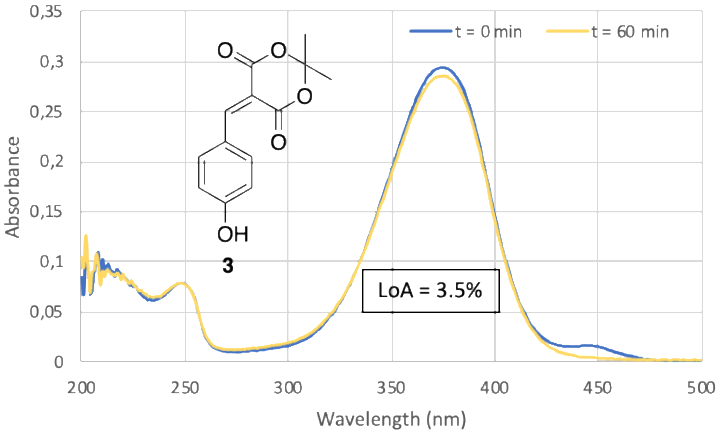

0,35
0,3
0,25
0,2
0,15
0,1
0,05
0
Absorbance
200
250
300
350
400
450
500
Wavelength (nm)
t = 0 min
t = 60 min
O
O
O
O
OH
3
LoA = 3.5%

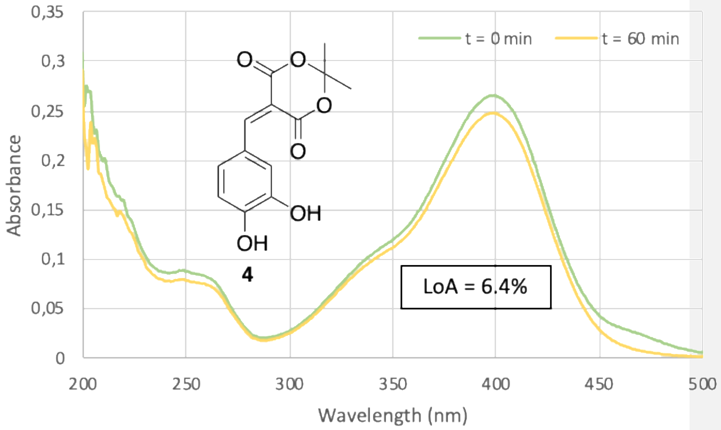

0,35
0,3
0,25
0,2
0,15
0,1
0,05
0
Absorbance
200
250
300
350
400
450
500
Wavelength (nm)
t = 0 min
t = 60 min
O
O
O
O
OH
OH
4
LoA = 6.4%

3.2. Furanic series

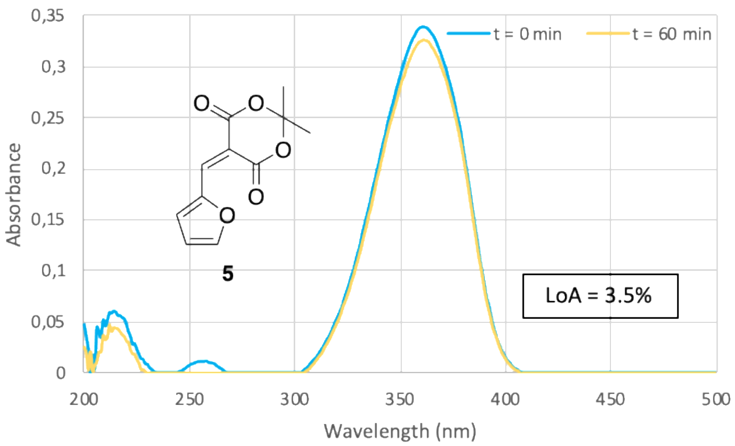

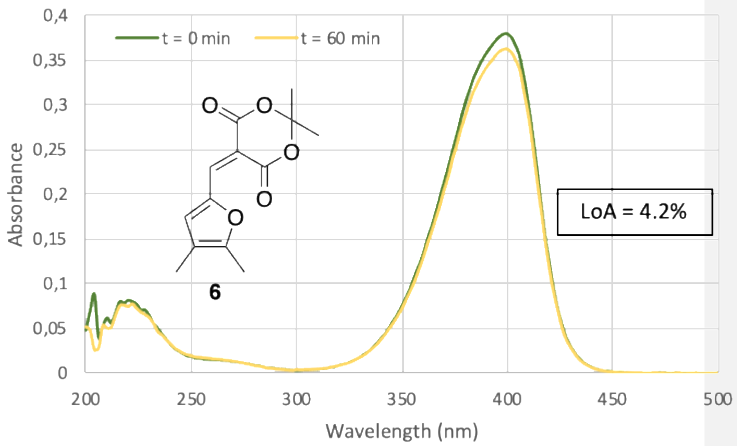

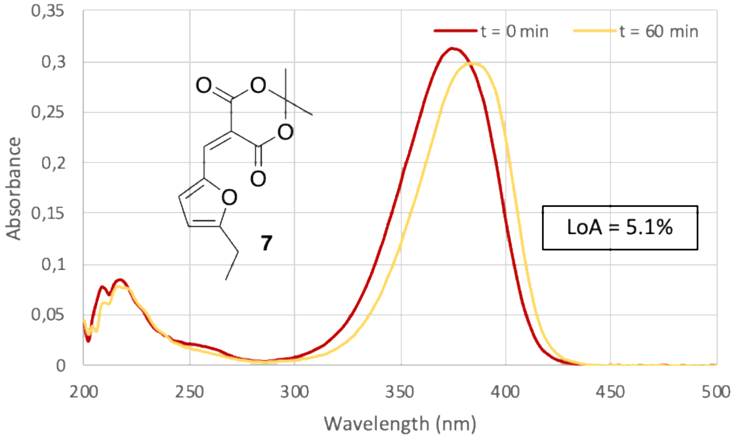
t = 0 min
t = 60 min
LoA = 5.1%
7
Absorbance
Wavelength (nm)

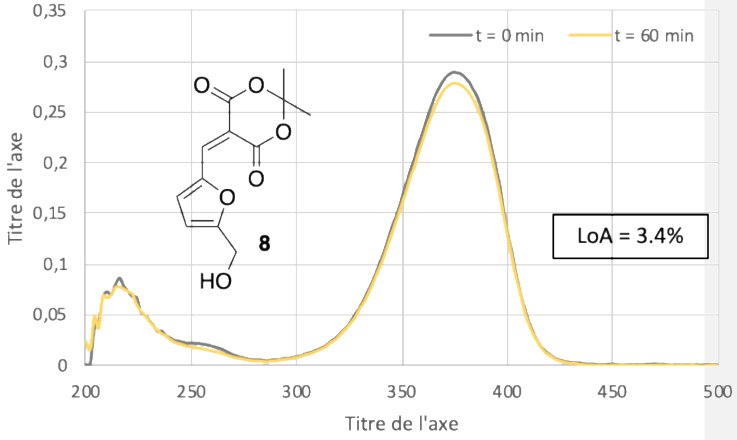
t = 0 min
t = 60 min
LoA = 3.4%
8
HO
Titre de l'axe
Titre de l'axe

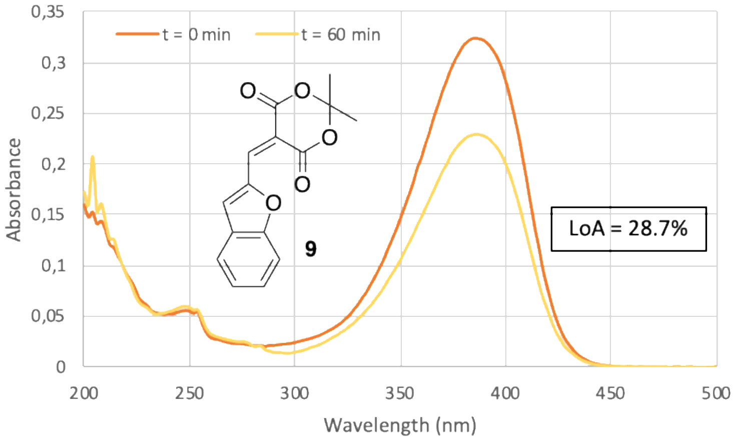
t = 0 min
t = 60 min
LoA = 28.7%
9
Absorbance
Wavelength (nm)

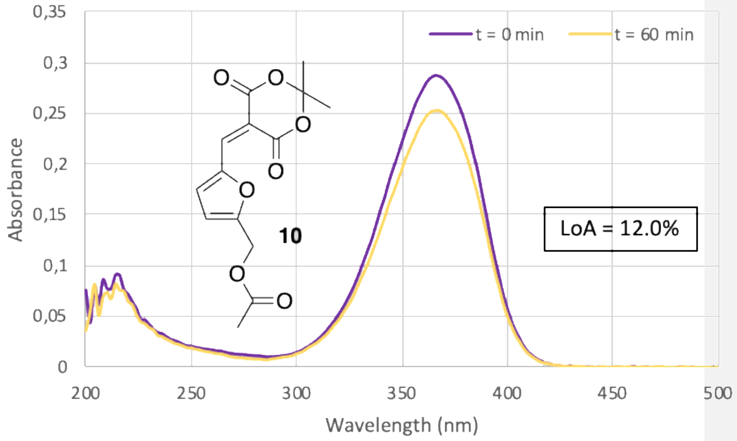
t = 0 min
t = 60 min
LoA = 12.0%
10
Absorbance
Wavelength (nm)

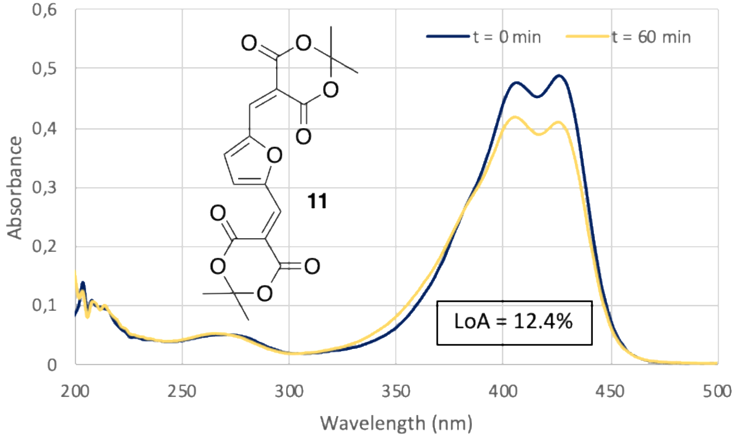

0,6
0,5
0,4
0,3
0,2
0,1
0
Absorbance
200
250
300
350
400
450
500
Wavelength (nm)
t = 0 min
t = 60 min
11
LoA = 12.4%

3.3. Pyrrolic series

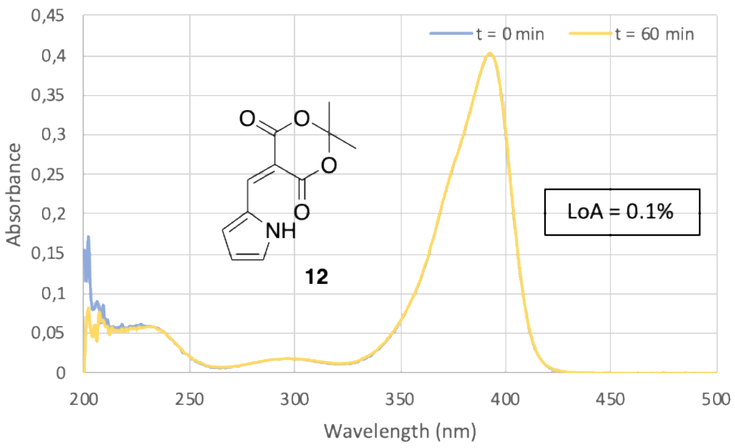

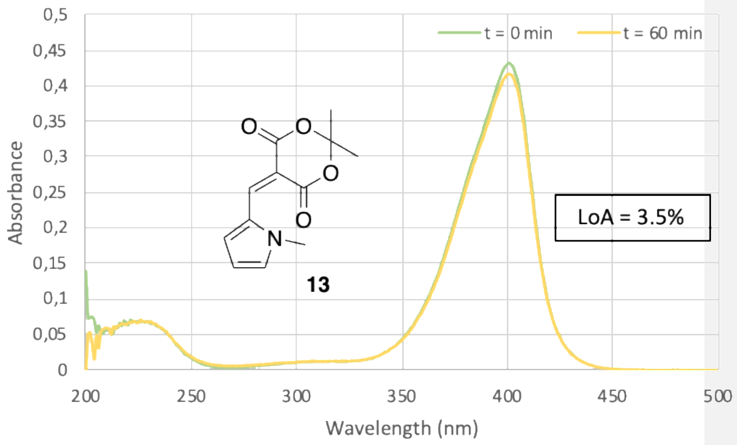

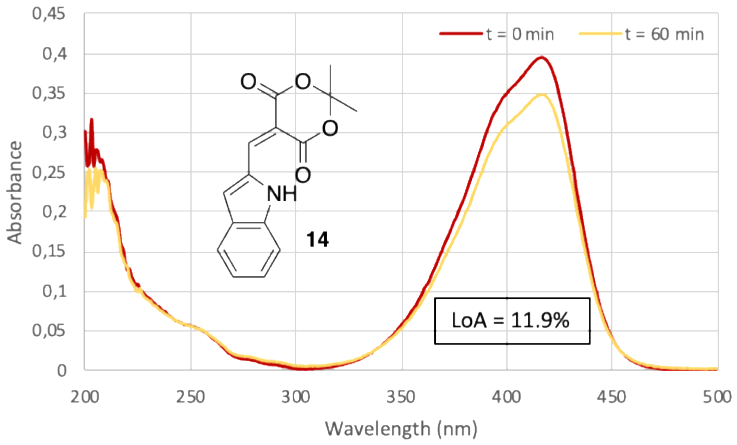

0,45
0,4
0,35
0,3
0,25
0,2
0,15
0,1
0,05
0
Absorbance
200
250
300
350
400
450
500
Wavelength (nm)
t = 0 min
t = 60 min
O
O
O
O
NH
14
LoA = 11.9%

3.4. Mixtures

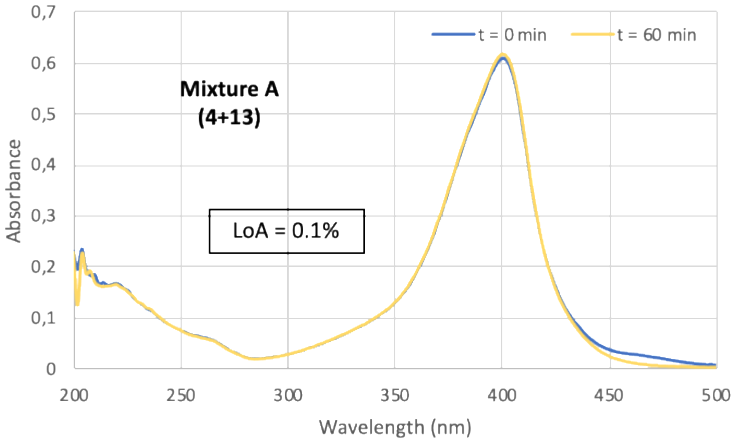

Mixture A
(4+13)
LoA = 0.1%
t = 0 min
t = 60 min
Absorbance
Wavelength (nm)
0,7
0,6
0,5
0,4
0,3
0,2
0,1
0
200
250
300
350
400
450
500

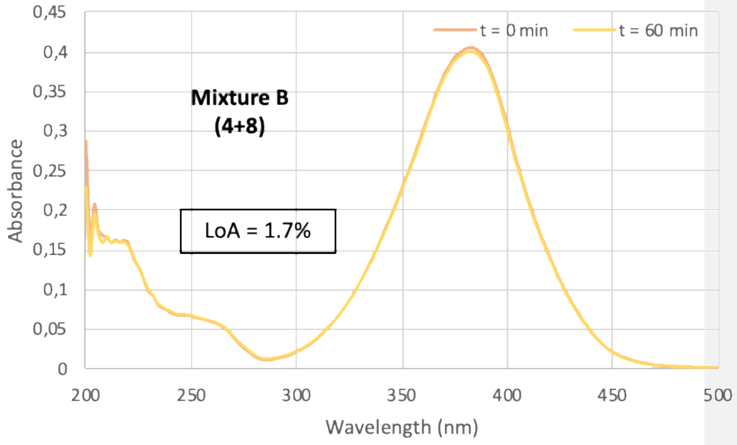

Mixture B
(4+8)
LoA = 1.7%
t = 0 min
t = 60 min
Absorbance
Wavelength (nm)
0,45
0,4
0,35
0,3
0,25
0,2
0,15
0,1
0,05
0
200
250
300
350
400
450
500

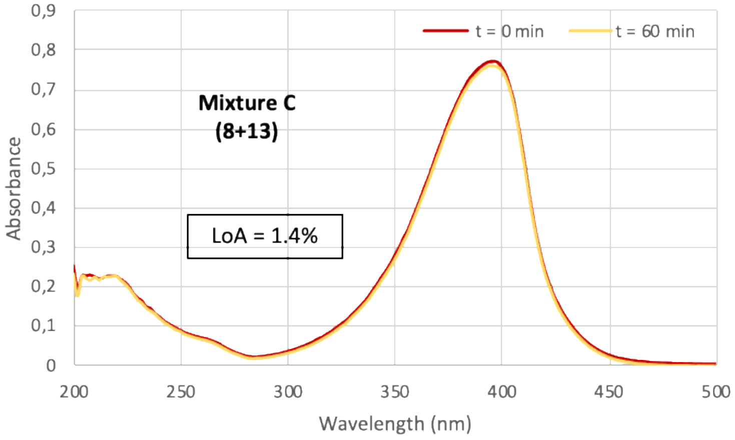
t = 0 min
t = 60 min
Mixture C
(8+13)
LoA = 1.4%
Absorbance
Wavelength (nm)
0
0,1
0,2
0,3
0,4
0,5
0,6
0,7
0,8
0,9
200
250
300
350
400
450
500

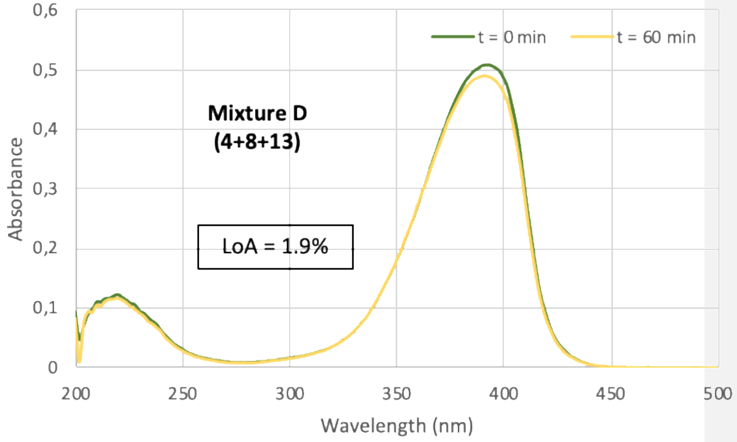
t = 0 min
t = 60 min
Mixture D
(4+8+13)
LoA = 1.9%
Absorbance
Wavelength (nm)
0
0,1
0,2
0,3
0,4
0,5
0,6
200
250
300
350
400
450
500

3.5. Reference

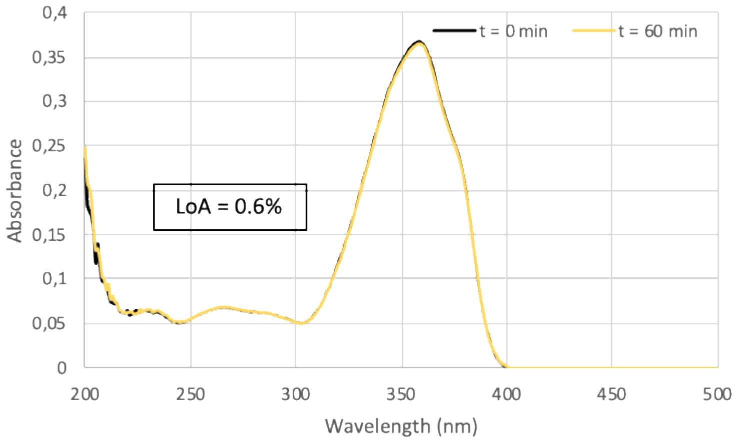

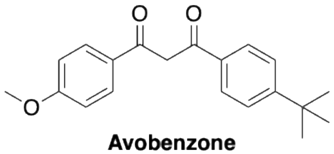

Avobenzone

4. DPPH assays

4.1. Phenolic series

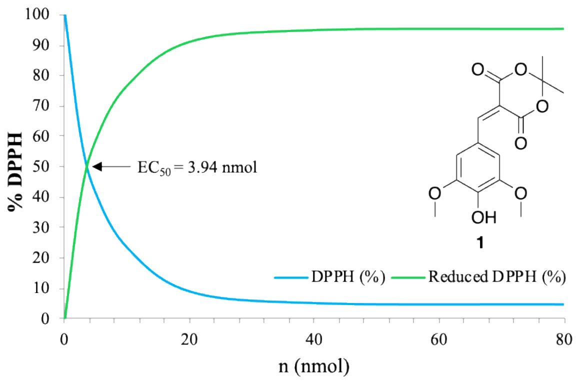

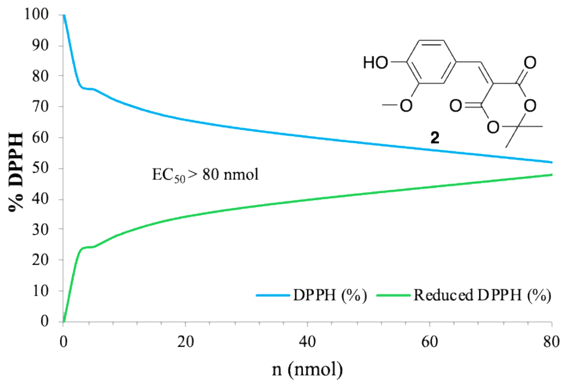

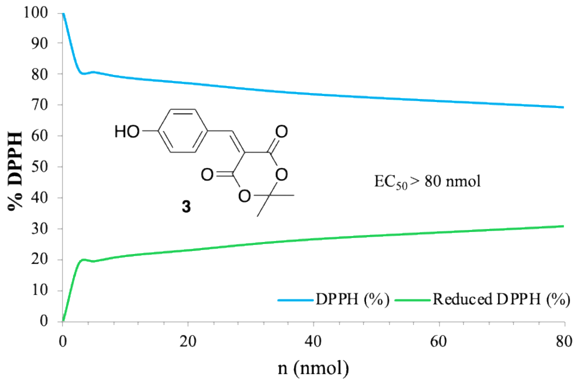

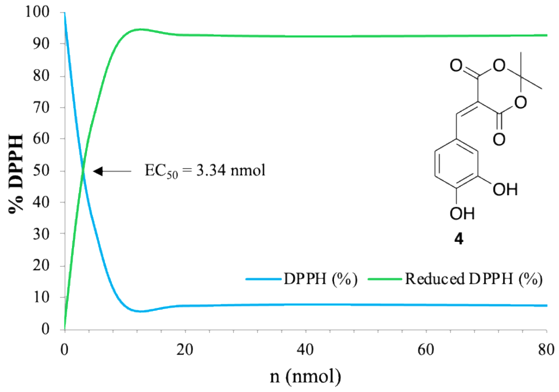

4.2. Furanic series

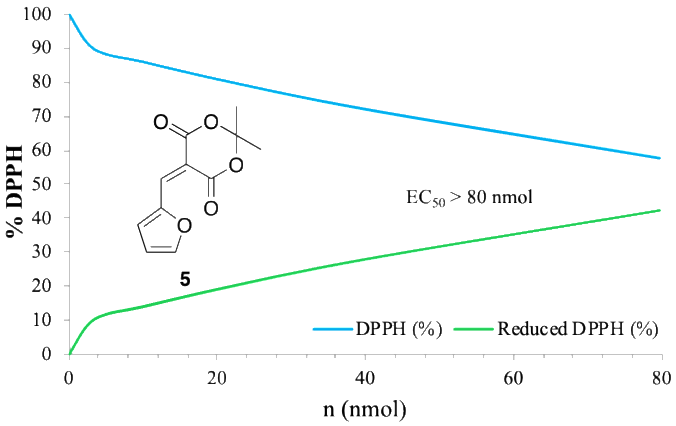

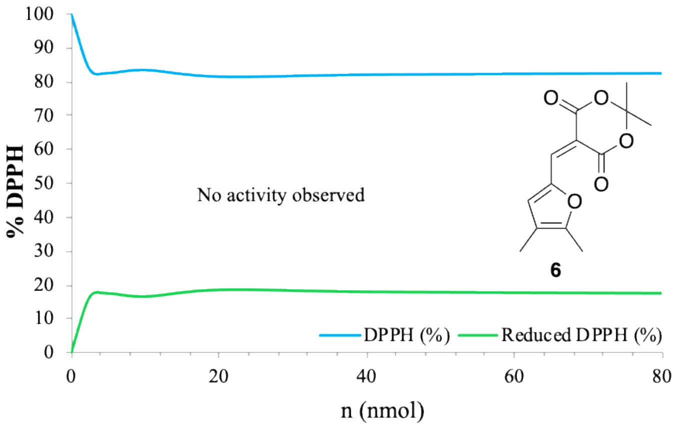

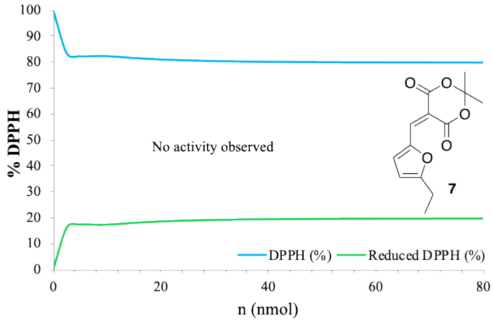
% DPPH
No activity observed
7
DPPH (%)
Reduced DPPH (%)
n (nmol)

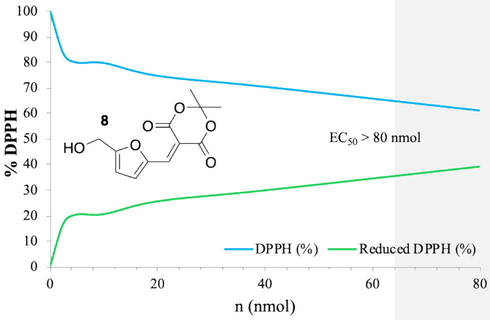
% DPPH
8
HO
EC50 > 80 nmol
DPPH (%)
Reduced DPPH (%)
n (nmol)

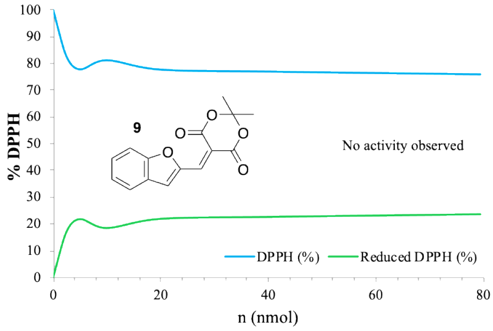
% DPPH
9
No activity observed
DPPH (%)
Reduced DPPH (%)
n (nmol)

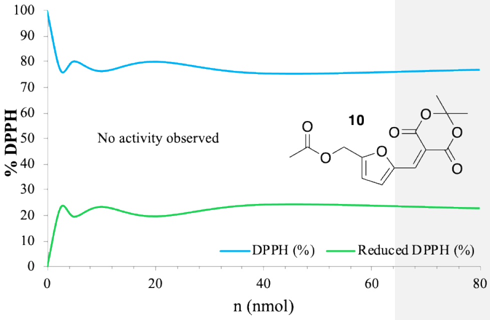
% DPPH
No activity observed
10
DPPH (%)
Reduced DPPH (%)
n (nmol)

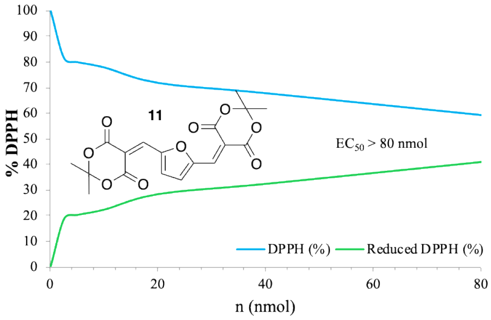
% DPPH
11
EC50 > 80 nmol
DPPH (%)
Reduced DPPH (%)
n (nmol)

4.3. Pyrrolic series

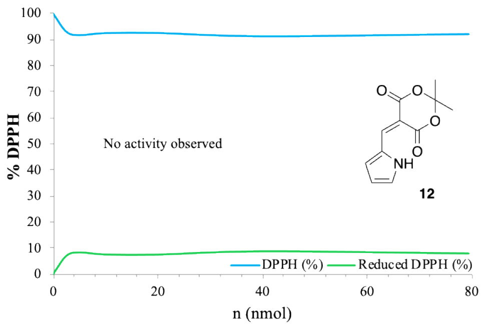

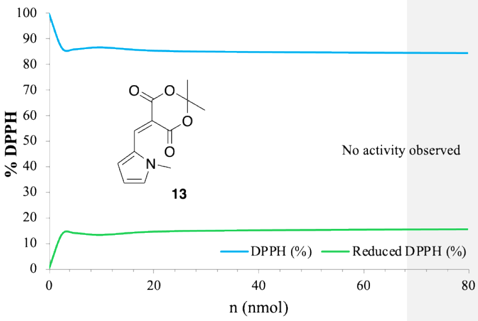

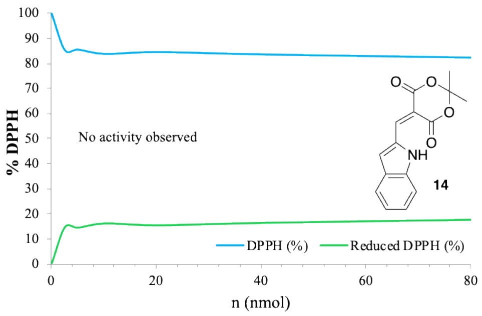

4.4. Mixtures

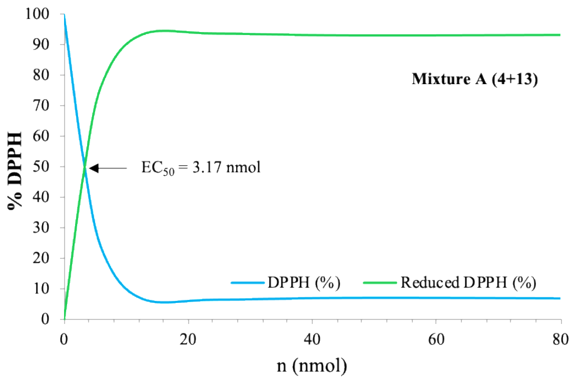

Mixture A (4+13)
EC50 = 3.17 nmol
DPPH (%)
Reduced DPPH (%)
% DPPH
n (nmol)

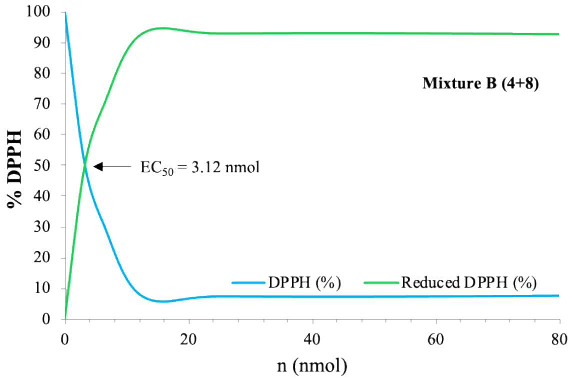

Mixture B (4+8)
EC50 = 3.12 nmol
DPPH (%)
Reduced DPPH (%)
% DPPH
n (nmol)

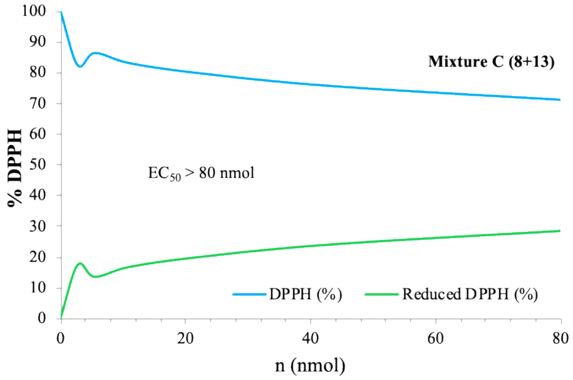

Mixture C (8+13)
EC50 > 80 nmol
DPPH (%)
Reduced DPPH (%)
% DPPH
n (nmol)

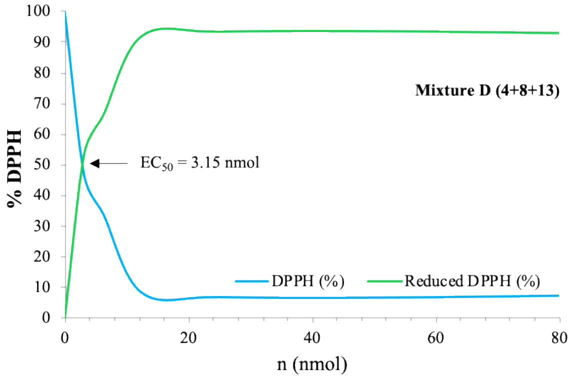

Mixture D (4+8+13)
EC50 = 3.15 nmol
DPPH (%)
Reduced DPPH (%)
% DPPH
n (nmol)

4.5. References

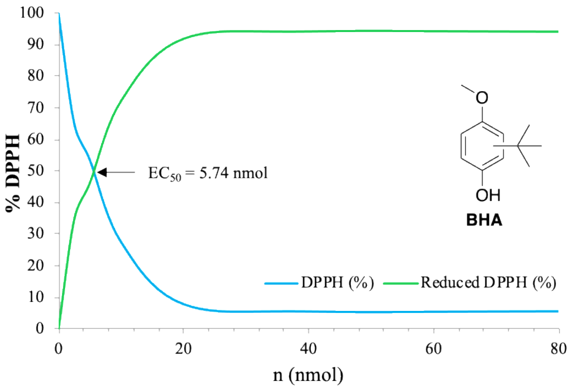

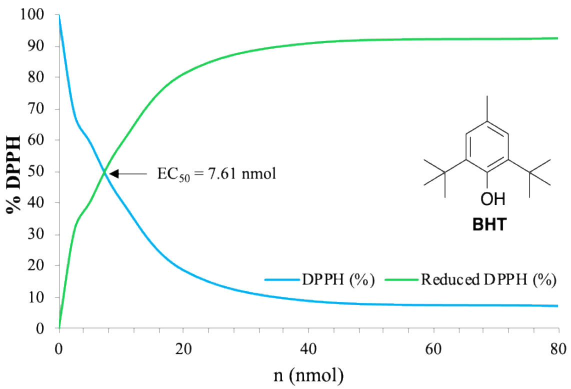

5. Tyrosinase inhibition

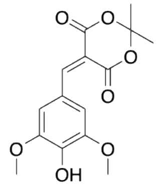

1

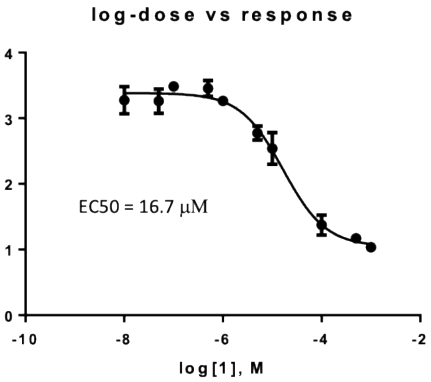

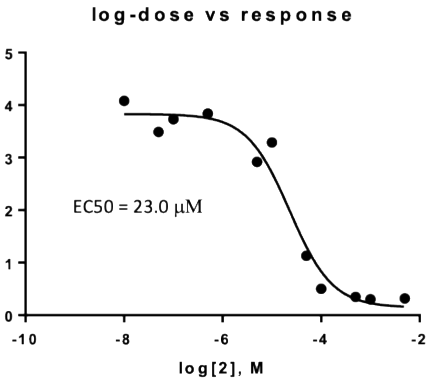

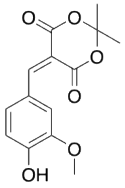

2

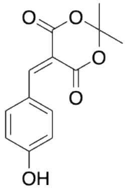

3

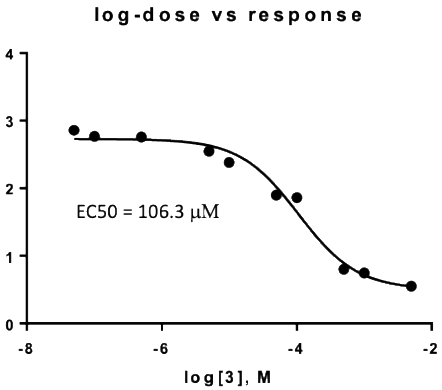

log-dose vs response
EC50 = 106.3 μM
log[3], M

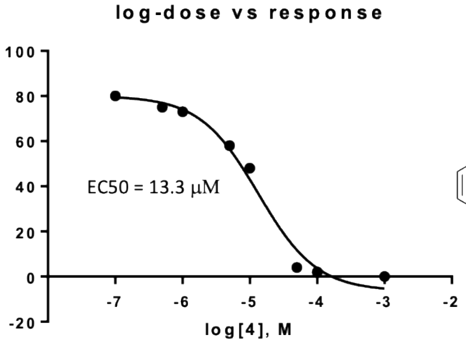

log-dose vs response
EC50 = 13.3 μM
log[4], M

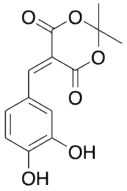

4

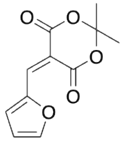

5

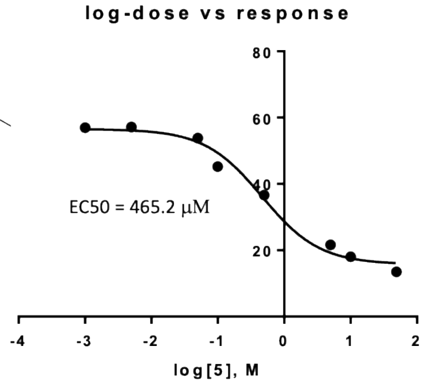

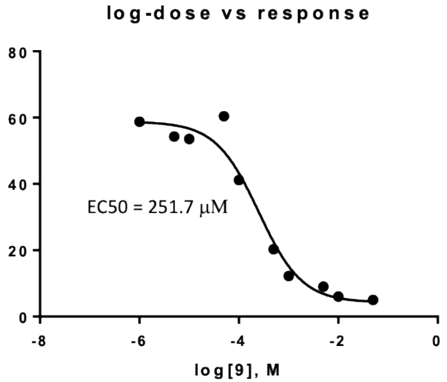

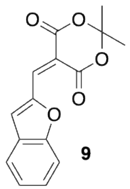

9

Mixture A (4+13)

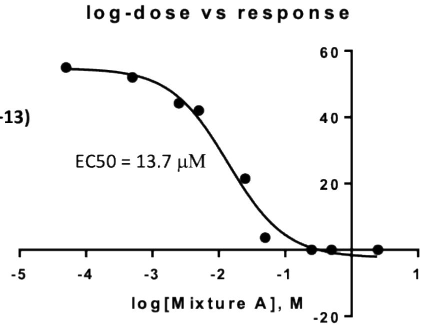

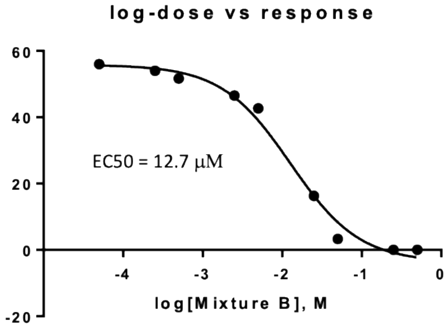

Mixture B (4+8)

Mixture D (4+8+13)

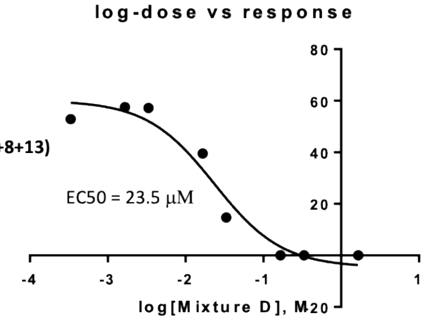

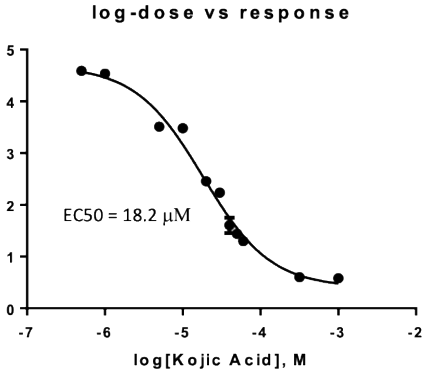

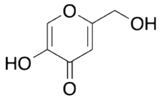

Kojic acid
